# The Skeletal-Protecting Action and Mechanisms of Action for Mood-Stabilizing Drug Lithium Chloride: Current Evidence and Future Potential Research Areas

**DOI:** 10.3389/fphar.2020.00430

**Published:** 2020-04-07

**Authors:** Sok Kuan Wong, Kok-Yong Chin, Soelaiman Ima-Nirwana

**Affiliations:** ^1^ Department of Pharmacology, Faculty of Medicine, Universiti Kebangsaan Malaysia, Kuala Lumpur, Malaysia; ^2^ State Key Laboratory of Oncogenes and Related Genes, Renji-Med X Clinical Stem Cell Research Center, Department of Urology, Ren Ji Hospital, School of Medicine, Shanghai Jiao Tong University, Shanghai, China

**Keywords:** bone, glycogen synthase kinase-3β, lithium, osteoblast, osteoclast, osteoporosis

## Abstract

Lithium, the lightest natural-occurring alkali metal with an atomic number of three, stabilizes the mood to prevent episodes of acute manic and depression. Multiple lines of evidence point to lithium as an anti-suicidal, anti-viral, anti-cancer, immunomodulatory, neuroprotective and osteoprotective agent. This review article provides a comprehensive review of studies investigating the bone-enhancing effects of lithium and its possible underlying molecular mechanisms. Most of the animal experimental studies reported the beneficial effects of lithium in defective bones but not in healthy bones. In humans, the effects of lithium on bones remain heterogeneous. Mechanistically, lithium promotes osteoblastic activities by activating canonical Wingless (Wnt)/beta (β)-catenin, phosphatidylinositol 3-kinase (PI3K)/protein kinase B (Akt) and bone morphogenetic protein-2 (BMP-2) transduction pathways but suppresses osteoclastic activities by inhibiting the receptor activator of nuclear factor-kappa B (RANK)/receptor activator of nuclear factor-kappa B ligand (RANKL)/osteoprotegerin (OPG) system, nuclear factor-kappa B (NF-κB), mitogen-activated protein kinase (MAPK), and calcium signaling cascades. In conclusion, lithium confers protection to the skeleton but its clinical utility awaits further validation from human clinical trials.

## Introduction

Bone-related conditions, such as osteoporosis, fracture and bone defect, impose an enormous burden on individuals and society in terms of incidence, prevalence and healthcare cost. The key to maintaining healthy bones is to attain an optimal peak bone mass during growth and reduce the rate of bone loss during adulthood (Bonjour et al., 2009). Some best practices for maintaining healthy bones include adopting a well-balanced diet, ensuring optimal intakes of calcium and vitamin D and performing weight-bearing physical activities. In terms of treatment for skeletal diseases, research on developing novel therapeutics and repurposing drugs are currently underway to identify drugs with greater efficacy but lesser side effects.

Lithium is a recognised treatment for bipolar disorder (Tondo et al., 2019). It helps to reduce the severity and frequency of acute mania and depression and stabilize mood between episodes (Gelenberg and Hopkins, 1993). Apart from that, maintenance lithium acts as a prophylaxis of recurrent mood disorders (Davis et al., 1999). Multiple assets of lithium have been explored beyond the mood-stabilizing effect such as anti-suicidal (Lewitzka et al., 2015), anti-viral (Zhai et al., 2019), anti-cancer (Li et al., 2014), immunomodulatory (Yang et al., 2019), neuroprotective (Forlenza et al., 2014), and skeletal-promoting properties (Zamani et al., 2009; Li et al., 2017a), which further substantiate the clinical usefulness of lithium. The latter effect is the main focus of this review.

Lithium is a specific inhibitor of glycogen synthase kinase-3 beta (GSK3β) *via* two important mechanisms whereby it directly inhibits GSK3β by competition with magnesium ions and indirectly inhibits GSK3β *via* serine phosphorylation (Eldar-Finkelman and Martinez, 2011). Since its discovery four decades ago as a protein kinase that phosphorylates and inhibits glycogen synthase, GSK3β has been demonstrated to be a point of convergence for multiple cell signaling pathways involved in physiological processes (Embi et al., 1980; Wang et al., 2011). For instance, GSK3β plays a functional role in Wingless (Wnt)/beta (β)-catenin, phosphatidylinositol 3-kinase (PI3K), and nuclear factor-kappa B (NF-κB) signaling pathways (Wang et al., 2011). Intriguingly, these signal transduction pathways have been implicated in the regulation of bone metabolism and homeostasis thus suggesting the concept of lithium as a potential osteoprotective agent.

The purpose of the current review is to provide data showing the bone-protecting effects of lithium in animals and humans. The potential mechanisms of action underlying its bone-sparing effects are also described. We hope to provide an overview of the effectiveness and efficacy of lithium against bone-related disorders to encourage its greater use of lithium apart from the established anti-manic property.

## Evidence Acquisition

The literature search was performed from November 15, 2019 until December 15, 2019 with PubMed and Medline electronic databases using query string “lithium AND (bone OR osteoporosis OR fracture OR osteoblast OR osteoclast OR osteocyte)”. The titles and abstracts were screened and relevant full-text articles were retrieved. A total of 40 original research articles inclusive of preclinical experimental evidence and human epidemiological data were included in this review.

## Effects of Lithium on Bone: Evidence From *In Vivo* Studies

The effects of lithium on bone have been widely established in various types of animals, including rodents, goats, rabbits, dogs, and chickens. The *in vivo* models utilised by investigators vary between studies, including the use of animals subjected to surgical castration, chemical castration, bone defects, and/or fractures, genetically senescence animals, knockout animals, as well as normal healthy animals (**Table 1**).

**Table 1:** Effects of lithium on bone *in vivo*.

Type of animal model	Method for induction of bone disorders	Treatment (dose, duration and mode of administration)	Findings	References
Female wild-type mice (Col1-Cre^-^ Cx43^flox/flox^)	Closed femur fracture	LiCl (200 mg/kg/day, 28 days of treatment, oral)	BV/TV: ↑, ultimate torque at fracture: ↑, torsional rigidity: ↑	(Loiselle et al., 2013)
Cx43 conditional knockout mice (Col1-Cre^+^ Cx43^flox/flox^)			BV/TV: ↑, ultimate torque at fracture: ↑, torsional rigidity: ↔	
Healthy female Sprague-Dawley rats	Closed midshaft unilateral femoral fracture	Lithium (20 mg/kg/day, 2 weeks of treatment, oral)	Maximum yield torque: ↑	(Bernick et al., 2014)
Female Sprague-Dawley rats	Closed right femoral diaphyseal fracture	LiCl (20 mg/kg, 2 weeks of treatment, oral)	Maximum yield torque: ↑	(Vachhani et al., 2018a)
Male Wistar rats	Bone defect (5 mm in length, 1.5 mm in width and 1 mm in depth) 6 mm below the knee joint	Bone defects were filled with BD Matrigel™ basement membrane matrix with Li_2_CO_3_ (10 mM, 14 days)	New BV/TV: ↔, percentage of lamellar bone volume: ↑, MAR: ↓, Ob.N: ↔, Oc.N: ↓	(Arioka et al., 2014)
Healthy adult male goats	Symmetrical round 10 mm bone defect at medialis face of tibia	Li-DBB scaffold	Callus has formed in defect region with excellent healing, calcified callus bone volume: ↑, Tb.Th: ↑, mean osteogenic area: ↑, Runx-2: ↑, COL1: ↑	(Guo et al., 2018)
Adult male Japanese white rabbits	Glucocorticoid-induced osteonecrosis and bone defects at femoral head	Bone defects were filled with Li-nHA	Moderate repair in bone defects, BV: ↑, bone density: ↑, new bone area: ↑, PI3K: ↔, Akt: ↔, GSK3β: ↓, β-catenin: ↑, PPAR-γ: ↓, ALP: ↑	(Li et al., 2018)
Healthy adult Japanese white rabbits	Bilateral tibia bone defect	Bone defects were filled with Li-CPP (1-2%)	Mature new bone interconnected and formed irregular bone trabeculae, new bone formation area: ↑	(Ma et al., 2019)
Female Sprague-Dawley rats	Ovariectomy-induced osteoporosis	LiCl (10 mM, 1st & 30th days after surgery, locally injected to distal femoral condyle)	BALP: ↑, PINP: ↓, TRACP-5b: ↓, CTX: ↓, BMD: ↑, BV/TV: ↑, SMI: ↓, Conn.D: ↑, BS/BV: ↔, Tb.N: ↑, Tb.Sp: ↓, Tb.Th: ↔	(Bai et al., 2019)
		LiCl + LY294002 (5 μM each, 1st & 30th days after surgery, locally injected to distal femoral condyle	BALP: ↑, PINP: ↓, TRACP-5b: ↓, CTX: ↓, BMD: ↑, BV/TV: ↑, SMI: ↓, Conn.D: ↑, BS/BV: ↓, Tb.N: ↑, Tb.Sp: ↓, Tb.Th: ↔	
Female Sprague-Dawley rats	Ovariectomy-induced osteoporosis and closed right femoral diaphyseal fracture	LiCl (20 mg/kg) – oral (treatment initiation on day 7)	Maximum yield torque: ↔, twist angle at failure: ↔, torsional stiffness: ↔	(Vachhani et al., 2018b)
		LiCl (20 mg/kg) – oral (treatment initiation on day 10	Maximum yield torque: ↑, twist angle at failure: ↔, torsional stiffness: ↑, better periosteal & mineralized callus bridging	
Female Sprague-Dawley rats	Ovariectomy-induced osteoporosis and bone defects at the medial aspect of the tibial midshaft	LiCl doped to CPC (50 or 100 mM, 8 weeks of treatment, implant)	BV/TV: ↑, gap between cement and bone was occupied entirely by new bone	(Li et al., 2017a)
Female Sprague-Dawley rats	Ovariectomy-induced osteoporosis and titanium implants inserted bilaterally into the proximal tibia	LiCl (150 mg/kg/2 days, 3 months of treatment, oral)	Trabeculae number & distribution: ↑, BIC: ↑, BV/TV: ↑, Tb.N: ↑, Tb.Th: ↑, Conn.D: ↑, OI: ↑, Tb.Sp: ↓, maximum push-out force: ↑, implant-bone interface shear strength: ↑	(Jin et al., 2017)
Male New Zealand white rabbits	Two holes (3 mm) were drilled at femoral shaft	Li-MAO-ETP (0.01 or 0.02 mol/L) – implant	Push-out load: ↑, bone ingrowth: ↑, bone mineralization: ↑	(Liu et al., 2018)
Male New Zealand white rabbits	Hole with 3.2 mm diameter were drilled at trabecular bone of the femur condyle	Li-SLA coated titanium discs – implant	Implant was covered with more osteoids, BV/TV: ↑, BIC: ↑	(Huang et al., 2019)
Male beagle dogs	–	LPPK – implant	BV/TV: ↑, Tb.N: ↑, Tb.Th: ↑, pull-out loads: ↑, BIC: ↑	(Zhang et al., 2018b)
Male Sprague-Dawley rats	Osteotomy and distraction osteogenesis	LiCl (200 mg/kg/day, 10 weeks of treatment, oral)	OCN: ↑, BV/TV: ↑, Tb.N: ↑, Tb.Th: ↑, Tb.Sp: ↑, BMD: ↑, stiffness: ↑	(Wang et al., 2015)
C57BL/6J mice	Titanium-induced calvarial osteolysis	LiCl (50 mg/kg/day, 14 days of treatment, i.p.)	BMD: ↑, BV/TV: ↑, number of pores: ↓, area of porosity: ↓, ES: ↓, Oc.N: ↓, percentage of osteoclasts: ↓, TNF-α: ↓, IL-1β: ↓, IL-6: ↓	(Hu et al., 2017)
		LiCl (200 mg/kg/day, 14 days of treatment, i.p.)		
Lrp5-knockout (*Lrp5^-/-^*) mice	–	LiCl (200 mg/kg, 4 weeks of treatment, oral)	BV/TV: ↑, Tb.Th: ↔, Tb.N: ↑, O.Th: ↑, Ob.N: ↑, Oc.N: ↔, Ad.N: ↓, MS: ↔, MAR: ↑, BFR: ↑, OCN: ↔, Dpyr: ↔, PTH: ↔, cortical BMD: ↔	(Clement-Lacroix et al., 2005)
SAMP6 mice			BV/TV: ↑, Tb.Th: ↑, Tb.N: ↑, O.Th: ↔, Ob.N: ↑, Oc.N: ↔, Ad.N: ↓, MS: ↔, MAR: ↑, BFR: ↑, Dpyr: ↓, cortical BMD: ↔	
C57BL/6 mice			BV/TV: ↑, Tb.Th: ↑, Tb.N: ↑, O.Th: ↑, Ob.N: ↑, Oc.N: ↔, MS: ↑, MAR: ↑, BFR: ↔, OCN: ↑, Dpyr: ↔, PTH: ↔, cortical BMD: ↔	
C57BL/6J mice	–	LiCl (10 mg/kg/day, 6 weeks of treatment, oral)	p-GSK3β: ↑, p-β-catenin: ↓, OPG: ↑, RANKL: ↔, OPG/RANKL ratio: ↑	(Kurgan et al., 2019)
Female Wistar rats	–	Li_2_CO_3_ (45 mg/kg/day, 3 months of treatment, oral)	Calcium: ↔, phosphorus: ↔, ALP: ↔, BV/TV: ↓, Tb.Th: ↔, Tb.N: ↓, Tb.Sp: ↑, thickness of growth plate cartilage: ↔, Ob.S: ↑, ES: ↑, Lc.S: ↓	(Lewicki et al., 2006)
Female Holtzman rats	–	LiCl (4 meq/kg, 4 weeks of treatment, i.p.)	Calcium: ↔, phosphorus: ↔, ALP: ↔, PTH: ↔, cortical bone area: ↔, medullary cavity area: ↔, periosteal & endosteal linear extent of bone mineralization: ↔, periosteal mineralization rate: ↓, endosteal mineralization rate: ↔, percent of matrix & surface occupied by osteoid: ↓	(Baran et al., 1978)
Male broiler chicks	–	LiCl (20 mg/kg, 3 or 5 weeks of treatment, oral)	Stiffness (femur): ↓, stiffness (tibia): ↔, energy to fracture (femur): ↔, energy to fracture (tibia): ↓, elastic modulus: ↔, yield & ultimate stress: ↔, load: ↔, toughness: ↔, BV/TV: ↔, Tb.Th: ↔, Tb.Sp: ↔, Tb.N: ↔	(Harvey et al., 2015)
Female Sprague-Dawley rats	–	LiCl (1.43 ± 0.13 meq/day, 40 days of treatment, oral)	Calcium: ↔, inorganic phosphorus: ↔, mineral salts: ↔, collagen hydroxyproline: ↔, neutral-salt-soluble collagen: ↔, resorption rate & formation rate of collagen: ↔, total incorporation of proline into bone matrix: ↔	(Henneman and Zimmerberg, 1974)
Female albino rats	–	Lithium (0.1 or 0.5 mM, 6 weeks of treatment, s.c.)	Calcium: ↔, magnesium: ↔, phosphorus: ↔, liver, skeletal muscle, femur mineral & bone histology: ↔	(Bellwinkel et al., 1975)

The bone-protecting effects of lithium have been evaluated in bone fracture models. Loiselle et al. (2013) created a closed femur fracture by three-point bending in two strains of mice, namely female wild-type mice (Col1-Cre^-^ Cx43^flox/flox^) and Connexin 43 (Cx43) conditional knockout mice (Col1-Cre^+^ Cx43^flox/flox^). Connexin 43, the most abundant gap junction protein in bone, is highly expressed during fracture healing as it helps in osteoblastic proliferation, differentiation and survival. The lack of Cx43 results in impaired bone formation and healing. The fractured animals were orally treated with lithium chloride (LiCl) at the dose of 200 mg/kg starting from day 4 post-fracture. They reported lower mineral apposition rate (MAR), bone formation rate (BFR), bone volume (BV)/total volume (TV), collagen type I alpha 1 (Col1α1), bone morphogenetic protein-2 (BMP-2), and osteocalcin (OCN) expressions in the Cx43 conditional knockout fractured mice compared to the wild-type fractured mice. Impaired osteoclastogenesis and functional deficits in healing were observed in the Cx43 conditional knockout fractured mice. Supplementation with LiCl rescued Cx43 conditional knockout fractures by increasing BV/TV, ultimate torque at failure and torsional rigidity (Loiselle et al., 2013). In another study, female Sprague-Dawley rats underwent closed midshaft unilateral femoral fractures using a blunt guillotine driven by a dropped weight. Fracture healing was maximized with oral administration of lithium (20 mg/kg) initiated seven days after fracture for 2 weeks. There was a 46% increment in maximum yield torque in the lithium-treated group compared to the non-treated controls (Bernick et al., 2014). In a recent study, closed diaphyseal fractures were performed on female Sprague-Dawley rats by dropping a ~300 g load on the right femur perpendicular to its longitudinal axis at the mid-shaft, followed by oral administration of LiCl for two weeks. The primary finding of this study was the higher maximum yield torque in the LiCl-treated animals than the vehicle-treated controls (Vachhani et al., 2018a).

Several investigators have demonstrated the protective effects of lithium on bone defects. By definition, bone defect refers to the structural loss of bone tissue where bone should normally exist. It is often sequelae of trauma, tumour or infection (Stewart et al., 2015). In an *in vivo* study, a bone defect (5 mm in length, 1.5 mm in width and 1 mm in depth) was made 6 mm below the knee joint of male Wistar rats and filled with BD Matrigel™ basement membrane matrix with lithium carbonate (Li_2_CO_3_, 10 mM) for 14 days. Micro-computed tomography (Micro-CT) analysis and bone histomorphometry were performed in the intracortical- and the endocortical-formation area. The osteoclast number (Oc.N) was significantly decreased but the percentage of lamellar BV was significantly increased, reiterating the acceleration of bone regeneration in promoting high-intensity bone formation (Arioka et al., 2014). In adult male goats, a symmetrical 10 mm round bone defect was introduced to the tibial facies medialis and the defect was filled with lithium-incorporated deproteinized bovine bone (Li-DBB) scaffold. Qualitatively, it was found that callus was formed in the defect region with dense and normal morphology of trabeculae in the Li-DBB group after 12 weeks. Quantitatively, it was noted that the mean gray values, mean pixel value, calcified callus BV, trabecular thickness (Tb.Th) and mean osteogenic area were significantly higher in bone defects filled with Li-DBB scaffold compared to those filled with deproteinized bovine bone (DBB) scaffold without lithium (Guo et al., 2018). In the same year, Li et al. (2018) examined the bone defect repairing effects of nano-lithium-hydroxyapatite (Li-nHA) scaffold in glucocorticoid-induced osteonecrosis of the femoral head in adult male Japanese white rabbits. Briefly, the rabbits were intravenously injected with lipopolysaccharide (LPS) followed by three intramuscularly injections of methylprednisolone acetate (20 mg/kg, time interval of 24 hours) into the right gluteus medius muscle after 24 hours. The femoral head defect was created and filled with Li-nHA scaffold. Micro-CT analysis showed that the Li-nHA group showed moderate defect repair, confirmed by the quantitative analysis expressed as higher values of bone volume and bone density as compared to the controls. Findings from histological detection also showed that the Li-nHA group presented a larger new bone area than the control animals (Li et al., 2018). More recently, lithium-doped calcium polyphosphate (Li-CPP) was fabricated by Ma and co-researchers to investigate its osteogenic potential. A bilateral tibial bone defect (5 mm in diameter and 3 mm in thickness) was built in adult male Japanese white rabbits and Li-CPP scaffold was implanted. Li-CPP scaffold was shown to enhance the formation of bone area. Histologically, there were mature interconnected new bones and irregular bone trabecular formed in the Li-CPP group (Ma et al., 2019).

Apart from that, the anti-osteoporotic effects of lithium have been evaluated *in vivo*. Ovariectomized rat model (the removal of both ovaries in female rat) remains the best validated animal model to mimic the clinical characteristics of postmenopausal osteoporosis in humans (Kalu, 1991). Using an osteoporosis rat model induced by ovariectomy, LiCl alone or in combination with LY294002 (a strong inhibitor of PI3K) was locally injected to the distal femoral condyle at the first and 30th days post-surgery. Serum level of bone alkaline phosphatase (BALP) was raised but the levels of procollagen type I N-terminal propeptide (PINP), tartrate-resistant acid phosphatase 5b (TRACP-5b) and C-terminal telopeptide of type 1 collagen (CTX) were lowered in the ovariectomized rats treated with LiCl alone. The combination of LiCl and LY294002 provided beneficial effects at a greater extent compared to LiCl alone. Similar outcomes were seen in the trabecular microarchitectural quantitative analysis of the femur in the ovariectomized rats, whereby the combination was better than the treatment alone (Bai et al., 2019). The ovariectomized rat model with fracture or bone defect was also used to assess the bone-sparing effects of lithium. Vachhani et al. (2018b) performed bilateral ovariectomy and a closed right femoral diaphyseal fracture in female Sprague-Dawley rats. Treatment with lithium at 20 mg/kg was initiated at day 10. The results indicated that lithium significantly improved healing at week 6 after fracture, as evidenced by higher maximum yield torque and torsional stiffness. Qualitatively, LiCl-treated femur displayed better periosteal and mineralized callus bridging (Vachhani et al., 2018b). In another study, a bone defect (diameter: 2.5 mm; depth: 4 mm) at the medial aspect to the tibial shaft was created after bilateral ovariectomy and it was filled with 50 or 100 mM LiCl doped to calcium phosphate cement (CPC). Data obtained from micro-CT showed more extensive newly-formed bone occurred and higher BV/TV at 8 weeks after the implantation of CPC with LiCl as compared to those without LiCl, indicating a better capacity of LiCl for bone regeneration (Li et al., 2017a). LiCl was also found to enhance implant osseointegration, implant fixation and bone regeneration in osteoporotic condition. In this study, female Sprague-Dawley rats underwent ovariectomy to induce osteoporosis and titanium was implanted in the tibia after 3 months. LiCl (150 mg/kg) was given to the rats every 2 days for 3 months. The positive outcomes observed in this study were increases in number and distribution of trabeculae, bone-implant contact (BIC), BV/TV, trabecular number (Tb.N), Tb.Th, connectivity density (Conn.D), osseointegration (OI), maximum push-out force, implant-bone interface shear strength, as well as a decrease in trabecular separation (Tb.Sp) in the LiCl-treated animals (Jin et al., 2017).

Recently, three groups of researchers delineated the effects of lithium on osteogenic integration using male New Zealand white rabbits and male beagle dogs. In an earlier study, Liu et al. (2018) fabricated an entangled titanium wire porous scaffold with a lithium-containing nanoporous coating (0.01 or 0.02 mol/L) by micro-arc oxidation (Li-MAO-ETP). Two holes (3 mm) were generated at the femoral shaft of rabbits and Li-MAO-ETP implants were inserted. The results indicated that the bone with Li-MAO-ETP implants had a higher push-out load, bone ingrowth and bone mineralization than the bone with MAO-ETP implants after 12 weeks of implantation (Liu et al., 2018). Huang et al. (2019) implanted lithium-incorporated sandblasted, large-grit, and acid-etched (Li-SLA) titanium discs into the trabecular bone of femur condyle in rabbits. In their study, osseointegration and new bone formation were assessed using micro-CT after 2, 4, and 8 weeks of implantation. More osteoids and new bone were covering the implants and higher BV/TV and BIC were detected in the Li-SLA group (Huang et al., 2019). Using a bigger animal model, Zhang and colleagues assessed the effects of lithium-doped silica nanospheres and poly(dopamine) composite coating on polyetheretherketone (LPPK) implantation on bone integration. The amount of newly formed bone tissues, BV/TV, Tb.N, Tb.Th, failure load and BIC in the LPPK group were higher compared to their respective controls (Zhang et al., 2018b).

In addition, the effects of lithium on distraction osteogenesis after osteotomy have been also evaluated. Distraction osteogenesis refers to a surgical process to stimulate new bone formation by cutting and gradually separating two bony fragments, allowing bone healing process between the gap (Neelakandan and Bhargava, 2012). Wang and co-researchers treated the rats subjected to osteotomy and distraction osteogenesis with LiCl (200 mg/kg/day) intragastrically. The LiCl-treated animals displayed higher OCN, BV/TV, Tb.N, Tb.Th, Tb.Sp, bone mineral density (BMD) and stiffness with more mature new bone tissue and bone fragments were completely fused in the distracted tibia (Wang et al., 2015). Using a mouse model of titanium particle-induced calvarial osteolysis, Hu et al. (2017) found that intraperitoneal (i.p.) injection of 50 or 200 mg/kg LiCl for 2 weeks increased BMD, BV/TV as well as decreased eroded surface (ES), Oc.N, pore number and area of porosity in mice calvaria (Hu et al., 2017).

In an *in vivo* study by Clement-Lacroix et al. (2005), LiCl was gavage-fed to three different strains of 8-week-old mice [low-density lipoprotein receptor-related protein 5 (Lrp5)-knockout (*Lrp5^-/-^*), senescence-accelerated mouse prone 6 (SAMP6) and C57BL/6 mice] and the effects of LiCl on bone metabolism were assessed after 4 weeks of therapy. The animals lacking the Lrp5 without LiCl treatment had reduced bone quality and treatment with LiCl improved the condition [evidenced by increased BV/TV, Tb.N, osteoid thickness (O.Th), osteoblast number (Ob.N), MAR, BFR, and reduced adipocyte number (Ad.N)]. Similar outcomes were detected in the other two mice strains treated with LiCl, whereby bone microstructure was improved and Ob.N was increased (Clement-Lacroix et al., 2005).

On the contrary, several groups of researchers reported negative or negligible effects of lithium on bone in healthy animals. In female Wistar rats, the animal receiving 45 mg/kg Li_2_CO_3_ in their drinking water for 3 months showed no significant differences in the serum levels of calcium, phosphorus and alkaline phosphatase (ALP). In comparison to the control animals, bone histomorphometric analysis indicated the reductions in BV/TV, Tb.N, and lining cell surface (Lc.S) as well as increases in Tb.Sp and ES in the Li_2_CO_3_-treated animals (Lewicki et al., 2006). Comparable outcomes were identified using healthy female Holtzman rats. Daily administration of LiCl (4 meq/kg, i.p.) for 4 weeks resulted in significant decreases in periosteal mineralization rate and volume of osteoid present in the tibia relative to the control animals. Levels of calcium, phosphorus, ALP, parathyroid hormone (PTH), and creatinine in serum were similar among the two experimental groups (Baran et al., 1978). In another study using growing broiler chickens as the animal model, oral supplementation of 20 mg/kg LiCl were initiated at 1 week or 3 weeks of age. The animals were euthanized at 6 weeks of age and bones were harvested for further analysis. Three-point bending test revealed that the LiCl-treated chickens had lower stiffness in the femur and lower energy to fracture in the tibia as compared to the controls. There were no alterations in parameters measured by micro-CT (Harvey et al., 2015). In one of the earlier studies, Henneman and Zimmerberg found that LiCl-supplemented drinking water (1.43 ± 0.13 meq/day) did not cause any changes in the serum electrolytes (total calcium and inorganic phosphorus), metaphyseal bone composition (calcium, collagen and mineral salts contents), and bone metabolism (amount of neutral-salt-soluble collagen, resorption rate of collagen, formation rate of collagen and total incorporation of proline into bone matrix) in female Sprague Dawley rats (Henneman and Zimmerberg, 1974). Moreover, Bellwinkel et al. (1975) reported no significant deviations in serum calcium, magnesium, and phosphorus levels of female albino rats after daily subcutaneous (s.c.) injection of a lithium-containing solution (0.1 or 0.5 mM) for 6 weeks. The calcium and magnesium content in liver, skeletal muscle and femur were also unaltered in rats by chronic lithium administration (Bellwinkel et al., 1975).

In summary, the accumulated *in vivo* evidence suggests the promising osteoprotective effects of lithium in defective bones and osteoporotic condition. However, lithium seems to have negligible or detrimental effects to healthy bones. Generally, lithium is administered to the animals *via* oral gavage, injections (i.p. or s.c.) or fabricated into scaffolds to be filled into the bone defects. Since lithium potentially helps in protecting bone *in vivo*, we would like to further look into the impact of lithium on bone in humans.

## Effects of Lithium on Bone: Evidence From Human Studies

The effects of lithium on bone have been investigated by researchers since decades ago but the findings are inconclusive. Some studies described the negative, negligible or positive effects of lithium on the skeletal system (**Table 2**). These human studies can be divided into epidemiology studies on the effects of lithium treatment in psychiatric patients, in which parameters related to bone were measured concomitantly and clinical trials specifically to study the effects of lithium on bone. Eren et al. (2006) pinpointed that treatment with Li_2_CO_3_ reduced BMD of the L2–L4 vertebrae and femur neck in the patients with bipolar disorder (n=15; aged 38.67 ± 8.23 years). Meanwhile, no changes were observed in the BMD of Ward’s triangle and femur trochanter in the Li_2_CO_3_-treated patients (Eren et al., 2006). Plenge and Rafaelsen (1982) assessed the effects of Li_2_CO_3_ treatment on calcium, magnesium, and phosphate balance as well as bone mineral content (BMC) in manic-melancholic patients. Two groups of patients were enrolled: (a) patients who were to start a Li_2_CO_3_ treatment and (b) patients who were to terminate a long-term Li_2_CO_3_ treatment. The excretion of calcium and phosphate were decreased but the excretion of magnesium was increased when treatment was initiated. Paradoxical outcomes were seen in patients who have terminated the treatment. However, the BMC of patients was found to decrease during the first 6 months of treatment. At that point, the authors could not clarify the positive balance of bone mineral and the slight demineralization observed in the patients (Plenge and Rafaelsen, 1982). In another study, 23 patients with affective disorders (women: aged 25–47 years; men: aged >25 years) were involved in the study conducted by Cohen et al. (1998). This study aimed to assess the short-term (0.4–1.0 year) or long-term (> 3 years) effects of Li_2_CO_3_ therapy on parameters of bone metabolism. There was no significant difference in all the bone-related blood and urine biochemical parameters as well as BMD between the two groups of subjects. The limitation of this study was the lack of longitudinal data, thus the comparison of bone metabolism parameters before and after treatment was not performed (Cohen et al., 1998). Another two-year prospective longitudinal study involving patients with bipolar affective disorder in manic phase or depressive phase and major depression (n=53, aged 16–63 years) showed that serum PTH level was significantly higher but creatinine clearance, phosphate excretion (24UPO_4E_), serum creatinine, ALP, albumin, inorganic phosphate (PO_4_), serum total calcium concentration adjusted for albumin (alb-adj Ca), and maximum tubular reabsorption of phosphate in relation to glomerular filtration rate (T_m_P/GFR) levels were not significantly different after 24 months of lithium therapy. However, the BMC and BMD of the subjects were not evaluated in this study (Mak et al., 1998). A nationwide population-based cohort study in Taiwan also found that bipolar disorder was associated with increased risk of fracture and the use of lithium [≥28 defined daily dose (DDD)] did not further increase the risk of fracture [hazard ratio (HR)=0.83; 95% CI 0.56–1.25] among subjects with bipolar disorder (n=3705; aged ≥16 years) (Su et al., 2017).

**Table 2:** Effects of lithium on bone in humans.

Type of subject	Treatment	Findings	References
Patients with bipolar disorders (n=15, aged 38.67 ± 8.23 years)	Li_2_CO_3_ (duration: 2 years)	Serum calcium & iPTH: ↑, serum phosphorus, T3, T4 & TSH: ↔, BMD (femur neck & L2-L4): ↓, BMD (femur trochanter & wards triangle): ↔	(Eren et al., 2006)
Manic-melancholic patients	Patients in neutral phase who were to start prophylactic Li_2_CO_3_ treatment	Calcium & phosphate excretion: ↓, magnesium excretion: ↑, BMC: ↓, Li_2_CO_3_ induced positive balance of calcium, magnesium and phosphate.	(Plenge and Rafaelsen, 1982)
	Patients who were on long term Li_2_CO_3_ treatment that was to be terminated	Calcium & phosphate excretion: ↑, magnesium excretion: ↔, Li_2_CO_3_ induced negative balance of calcium, magnesium and phosphate.	
Patients with affective disorders (n=23, women: aged 25-47 years; men: aged >25 years)	Li_2_CO_3_ (less than 12 months)	Serum creatinine, calcium, phosphate, ALP, thyroxin, TSH & PTH: ↔, calcium, cortisol & hydroxyproline excretion: ↔, BMD (lumbar spine & proximal femur): ↔.	(Cohen et al., 1998)
	Li_2_CO_3_ (more than 3 years)		
Patients with bipolar disorders or major depression (n=53, aged 16-63 years)	Lithium (duration: 2 years)	Serum PTH: ↑, creatinine clearance, 24UPO_4E_, serum creatinine, ALP, albumin, PO_4_, alb-adj Ca, & T_m_P/GFR: ↔.	(Mak et al., 1998)
Subjects with bipolar disorder (n=3705; aged ≥16 years)	Lithium (< 28 DDD or ≥28 DDD)	Fracture risk: ↔.	(Su et al., 2017)
Patients with maniac-depressive psychosis (n=26; women: aged 53 ± 10 years, men: aged 48 ± 9 years)	Lithium (duration: ≥10 years)	Ionized calcium concentration: ↑, BMD (lumbar spine): ↑, serum PTH: ↔.	(Nordenstrom et al., 1994)
Subjects (including children) with any fracture sustained during year 2000 (n=124655; aged 1-100 years)	Lithium use (< 250 DDD, 250–849 DDD or ≥850 DDD)	Fracture risk: ↓	(Vestergaard et al., 2005)
Patients with first record of any fracture during General Practice Research Database (GPRD) follow-up (n=231778; aged ≥18 years)	Current, recent and past lithium users (serum lithium level not measured)	Current users fracture risk: ↓, Past users fracture risk: ↑.	(Wilting et al., 2007)
Patients with osteoporotic fracture (n=15792; aged ≥50 years)	Current use of lithium (defined as at least one dispensation within 120 days preceding the index date of the fracture)	Fracture risk: ↓	(Bolton et al., 2008)
Subjects on lithium maintenance therapy for at least one year (n=75; aged 37 ± 9.6 years)	Serum lithium level = 0.8 ± 0.13 mmol/L	Bone density (spine, femoral neck & trochanter): ↑, ALP: ↓, CTX: ↓, OCN: ↓, total calcium: ↔, PTH: ↔, urinary calcium excretion: ↔	(Zamani et al., 2009)
Patients with mental disorders (n=68730; aged 64.2 ± 11.2 years)	Lithium use	Major osteoporotic fracture risk: ↓	(Bolton et al., 2017)

The protective effects of lithium on skeleton have also been demonstrated by researchers. A cross-sectional study performed by Nordenstrom et al. (1994) aimed to investigate the effects of long term lithium treatment (≥10 years) on PTH, ionized and total calcium levels in patients with manic-depressive psychosis (n=26; women: aged 53 ± 10 years, men: aged 48 ± 9 years). Higher ionized calcium level, BMD for the whole body, lumbar spine, and femoral neck but not significantly higher PTH level were detected in lithium-treated patients in relative to the normal healthy subjects (Nordenstrom et al., 1994). Vestergaard et al. (2005) designed a case-control study to examine fracture risk among users of lithium. Subjects with any fracture sustained during the year 2000 (n=124,655; aged 1–100 years) and their matching controls (n=373,962; aged 1–100 years) were recruited. Findings from the study found that lithium treatment was associated with decreased risk of any fracture [individual with 250–849 DDD of Li: odd ratio (OR)=0.74; 95% confident interval (95% CI): 0.60–0.92; individual with ≥850 DDD of Li: OR=0.67; 95% CI: 0.55–0.81]. Significant decreases in Colles’ (OR=0.57; 95% CI: 0.35–0.94) and spine fractures (OR=0.32; 95% CI: 0.11–0.95) were also observed in individuals with ≥850 DDD of lithium (Vestergaard et al., 2005). Another case-control study was performed within the United Kingdom General Practice Research Database to compare the use of lithium among patients with a sustained fracture and their matched controls. The results indicated that the current users of lithium (defined as those who received a lithium prescription within 2 months before the index date) had a decreased risk of fractures. Meanwhile, past users of lithium (defined as those who received the last lithium prescription more than 12 months prior to index date) had an increased risk of fractures, increasing with time since discontinuation. However, there was no association between fracture risk and cumulative duration of lithium use (Wilting et al., 2007). Subsequently, a case-control study reported that current lithium use was associated with a lower likelihood of osteoporotic fracture (OR=0.63; 95% CI 0.43–0.93) among individuals aged 50 years and above (n=15,792) (Bolton et al., 2008). In a study conducted by Zamani et al. (2009), patients on lithium maintenance therapy for more than one year (n=75; aged 37 ± 9.6 years) were enrolled to assess the effects of lithium on bone mass. The compliance of lithium therapy was assured by serum lithium level in the recommended therapeutic range (0.6–1.2 mmol/L). Results obtained from this study showed that bone density at the spine, femoral neck and trochanter of lithium-treated patients was higher than those in the controls. The serum ALP, CTX, and OCN were significantly lower in the lithium-treated patients as compared to the controls whereas the total calcium, PTH and urinary calcium excretion were not statistically different between the two groups (Zamani et al., 2009). In Canada, a cohort study involving 68730 individuals (62275 women and 6445 men; aged 64.2 ± 11.2 years) showed that lithium medication was associated with reduced risk of major osteoporotic fracture (HR=0.82; 95% CI 0.46–1.44) (Bolton et al., 2017).

Among the human studies included in this review, seven studies reported positive effects of lithium whereas other studies showed negative or negligible outcomes on the bone. The distinct outcomes observed in these human studies might be due to variations of study design, study population, study duration, population size, treatment duration and measurement of bone parameters. In most of the studies included, the dose of lithium prescribed to the patients was not mentioned. Therefore, it is challenging to speculate the optimum dose of lithium on bone whether it is higher, same or lower than the dose for psychiatric disorders. Recently, a comprehensive systematic review and meta-analysis was done to explore the effects of lithium on BMD and fracture risk using seven observational studies (Nordenstrom et al., 1994; Vestergaard et al., 2005; Wilting et al., 2007; Bolton et al., 2008; Zamani et al., 2009; Bolton et al., 2017; Su et al., 2017). The results indicated that lithium medication was significantly associated with a 20% decrease in the risk of fractures (Liu et al., 2019). In view of the beneficial effects of lithium on bone in the majority of the studies with the exception in few studies, further discussion pertaining the effects of lithium on bone cells *in vitro* and the understanding of its mechanisms of action is indispensable.

## The Underlying Molecular Mechanisms of Lithium in Protecting Bone Health

The integrity of the skeleton is maintained by the balanced activities of new bone formation by osteoblasts and resorption of mineralized bone matrix by osteoclasts. Bone formation (also known as osteogenesis or ossification) is a multi-step process beginning with the proliferation of progenitor cells into osteoblastic lines, followed by osteoblast differentiation, matrix maturation and mineralization (Setiawati and Rahardjo, 2018). Markers that are associated with osteoblast differentiation and bone formation include type I collagen (COL1), ALP, OCN, bone sialoprotein (BSP), osterix (OSX), and runt-related transcription factor 2 (Runx-2) (Chapurlat and Confavreux, 2016; Wong et al., 2019). On the other hand, bone resorption is a process of breaking down bone tissues by osteoclasts releasing minerals into the bloodstream. Osteoclastogenesis (osteoclast formation) is a complex sequential process that requires the commitment of pluripotent haematopoietic stem cells and its proliferation towards osteoclast precursors mediated by macrophage-colony stimulating factor (M-CSF) as well as the differentiation of osteoclast precursors into multinucleated and mature cells mediated by receptor activator of nuclear factor-kappa B ligand (RANKL) (Yavropoulou and Yovos, 2008). Mature osteoclasts attach themselves to the bone surfaces, followed by secretion of hydrochloric acid and cathepsin K (CTSK, a matrix-degrading collagenase) to resorb bone effectively (Boyce et al., 2009). Osteoclast number, resorption pit area, and tartrate-resistant acid phosphatase (TRAP) activity are often used to assess the bone resorption ability of osteoclasts (Bai et al., 2019). *In vitro* studies on the effects of lithium on bone cells have focused on its capability in modulating osteoblastogenesis and osteoclastogenesis (**Table 3**).

**Table 3:** Effects of lithium on bone cells *in vitro*.

Type of bone cell	Treatment	Findings	References
Murine osteoblast precursor (C3H10T1/2) cells	LiCl (10 mM)	ALP activity: ↑, p-β-catenin: ↓, Runx-2: ↑	(Arioka et al., 2014)
Primary osteoblasts from calvariae of 3-day-old rats	LiCl (10 mM)	p-Akt: ↔, p-GSK3β: ↑, p-β-catenin: ↓, NFATc1: ↓, ALP activity: ↑	(Bai et al., 2019)
	LiCl (5 mM) + LY294002 (5 μM)	p-Akt: ↑, p-GSK3β: ↑, p-β-catenin: ↓, NFATc1: ↓, ALP activity: ↑	
Calvaria cells recovered from Lrp5-knockout mice	LiCl (20 mM)	Percent of apoptotic calvarial osteoblast cells: ↓, cell differentiation into adipocyte lineage: ↓	(Clement-Lacroix et al., 2005)
MSCs derived from Lrp5-knockout mice		ALP activity: ↑, COL1α1: ↑	
Rat bone marrow MSCs stimulated with titanium particles	LiCl (1 or 5 mM)	ALP: ↑, extracellular matrix mineralization: ↑	(Yang et al., 2019)
Bone marrow MSCs from male Sprague-Dawley rats	Li-SLA coated titanium discs	ALP: ↑, OCN: ↑, COL1: ↑, Runx-2: ↑, BMP-2: ↑, β-catenin: ↑, OPG: ↑, RANKL: ↔	(Huang et al., 2019)
Bone marrow MSCs	Li-nHA	ALP-positive cells: ↑, calcium nodule: ↑, COL1: ↑, formation of lipid: ↓, p-GSK3β: ↑, β-catenin: ↑, Runx-2: ↑, Akt: ↑, PPAR-γ: ↓	(Li et al., 2018)
Murine pluripotent mesenchymal (C2C12) cells	Li-SLA coated titanium discs (1 mM)	ALP: ↑, OPG: ↑	(Galli et al., 2013)
Murine calvaria MC3T3 cells		OPG: ↑, OCN: ↑	
Primary murine bone marrow cells		ALP: ↑, CTGF: ↑, Wisp2: ↑, Cox-2: ↑, OPG: ↔, Runx-2: ↔, COL1α1: ↔, OCN: ↑, ALP activity: ↑	
Rat bone marrow MSCs	Lithium-doped mesoporous silica nanospheres (0.3–0.5 mg/mL)	Cell proliferation: ↑, ALP activity: ↑, Runx-2: ↑, ALP: ↑, OCN: ↑, OPN: ↑	(Zhang et al., 2018a)
Bone marrow stromal cells from adult Sprague-Dawley rats	LPPK	Cell proliferation: ↑, ALP activity: ↑	(Zhang et al., 2018b)
MC3T3-E1 cells	LiCl (50 or 100 mM)/CPC extract	Cell proliferation: ↑, ALP activity: ↑, osteogenic mineralization: ↑, spreading & extension of filopodia: ↑, COL1α1: ↑, OCN: ↑, OPG: ↑, Runx-2: ↑, p-GSK3β: ↑, p-β-catenin: ↓	(Li et al., 2017a)
Human osteosarcoma (MG63) cells	LiCl (50 or 100 mM)/CPC extract	Cell attachment and growth were supported, cell proliferation: ↑, ALP activity: ↑	(Li et al., 2017b)
Human osteosarcoma (MG63) cells	Li-DBB scaffold	Cell proliferation activities: ↑, calcium deposition: ↑	(Guo et al., 2018)
Human osteosarcoma (MG63) cells	Li-MAO-ETP scaffold	Cell viability: ↑, ALP activity: ↑, OPN: ↑, OCN: ↑, COL1α1: ↑, Runx-2: ↑, LRP5: ↑, LRP6: ↑, Axin2: ↑, β-catenin: ↑, p-β-catenin: ↓, p-GSK3β: ↑	(Liu et al., 2018)
Human osteosarcoma (MG63) cells	Li-CPP scaffold (2%)	Cell viability: ↑, GSK3β: ↓, β-catenin: ↑, Runx-2: ↑, ALP: ↑, mineralized nodule: ↑, calcium deposition: ↑	(Ma et al., 2019)
Murine pre-osteoblastic (MC3T3-E1) cells	LiCl (10 mM)	Mineral deposition: ↓, cell proliferation: ↑, ALPL activity: ↓, Runx-2: ↓, OSX: ↓, Alpl: ↓, OCN: ↓, p-SMAD1/5/8: ↓, p-p38: ↔, p-ERK1/2: ↔	(Li et al., 2011)
Mouse primary osteoblasts		Expression of *Runx-2*, *OSX*, *Atf4*, *Dlx5*, *Alpl*, *COL1α1*, *Ibsp*, *Spp1* and *OCN* at day 3: ↓, the inhibited osteoblast differentiation was diminished at day 6 and completely abolished at day 9.	
Murine pluripotent mesenchymal (C2C12) cells		Runx-2: ↓, OSX: ↓, Alpl: ↓, OCN: ↓, p-SMAD1/5/8: ↓, p-p38: ↔, p-ERK1/2: ↔, SMAD6: ↓, SMAD7: ↓	
Murine osteoclast precursor (RAW-D) cells treated with RANKL	LiCl (10 mM)	TRAP activity: ↓, TRAP-positive multinucleated cells: ↓, NFATc1: ↓	(Arioka et al., 2014)
Bone marrow macrophages treated with M-CSF and RANKL	LiCl (10 mM)	p-Akt: ↔, p-GSK3β: ↑, p-β-catenin: ↓, NFATc1: ↓, pit area: ↓, TRAP: ↓	(Bai et al., 2019)
	LiCl (5 mM) + LY294002 (5 μM)	p-Akt: ↑, p-GSK3β: ↑, p-β-catenin: ↓, NFATc1: ↓, pit area: ↓, TRAP: ↓	
RAW264.7 macrophages stimulated with titanium particles	LiCl (1 or 5 mM)	TNF-α: ↓, IL-6: ↓, IL-4: ↑, IL-10: ↑, BMP-2: ↑, VEGF: ↑, p-ERK: ↓, p-p38: ↓	(Yang et al., 2019)
Bone marrow macrophages treated with M-CSF and RANKL	Li-SLA coated titanium disc	Oc.N: ↓	(Huang et al., 2019)
Bone marrow macrophages treated with M-CSF and RANKL	LiCl (0.2–5 mM)	Oc.N: ↓, area of osteoclast per well: ↓, bone resorption area: ↓, CTSK: ↓, Oscar: ↓, Sema-4A: ↓, p-GSK3β: ↑, p-Akt: ↔, p-ERK: ↔, p-p38: ↔, p-JNK: ↔, p-IκBα: ↓, p-p65: ↓, c-Fos: ↓, NFATc1: ↓	(Hu et al., 2017)
Foetal long bones stimulated for resorption by PTH, PGE_2_, IL-1 or 1,25(OH)_2_D_3_	Lithium (3 mM)	No effect on basal resorption and resorption stimulated by IL-1; slight and inconsistent inhibitory effect on resorption stimulated by PTH; moderate and inconsistent inhibitory effect on resorption stimulated by PGE_2_; complete inhibition of resorption by 1,25(OH)_2_D_3_.	(Pepersack et al., 1994)

### Effects of Lithium on Osteoblastogenesis

Bone marrow-derived mesenchymal stem cells (MSCs) are progenitor cells capable of proliferation and differentiation into mature osteoblasts (Zomorodian and Baghaban Eslaminejad, 2012; Dai et al., 2015). Incubation of osteoblast precursor cells or mature osteoblasts with LiCl stimulated markers of osteoblast differentiation (as shown by increased ALP, COL1, and Runx-2) and enhanced calcium deposition (as shown by increased Alizarin red staining) (Clement-Lacroix et al., 2005; Arioka et al., 2014; Li et al., 2018; Bai et al., 2019; Yang et al., 2019). Two studies measured the osteogenic effects of Li-SLA treated titanium discs by seeding them with murine bone marrow mesenchymal stem cells (MSCs) or osteoblastic MC3T3 cells. As compared to the SLA group, Li-SLA treated titanium discs was beneficial for osteoblastic proliferation (assessed by higher expression of ALP, OCN, COL1, and Runx-2) (Galli et al., 2013; Huang et al., 2019). In another study, Zhang J. et al. (2018a) cultured rat bone marrow MSCs with lithium-doped mesoporous silica nanospheres. The release of lithium ions from lithium-doped mesoporous silica nanospheres enhanced the expression of osteogenesis-related genes, such as Runx-2, ALP, OPN, and OCN (Zhang et al., 2018a). The same group of researchers also reported significant stimulatory effects of LPPK on cell proliferation and ALP activity of rat bone marrow stromal cells (Zhang et al., 2018b). A trace amount of lithium doped onto CPC has demonstrated the characteristics of enhanced osteoblastic proliferation, and differentiation, shown by increased ALP activity, osteogenic markers (COL1α1, OCN, OPG, and Runx-2), and mineralization (Li et al., 2017a). Using human osteosarcoma (MG63) cells, several studies in recent years also reported that lithium had promising effects on osteoblast proliferation and differentiation. Lithium-doped CPC had high biocompatibility, supported osteoblast attachment and growth, and promoted cell proliferation and differentiation in MG-63 cells (Li et al., 2017b). It was noteworthy that MG-63 cells cultured on bone substitute coated with lithium had higher cell viability, cell proliferation, cell differentiation, calcium deposition, and expression of osteogenesis-related genes (Guo et al., 2018; Liu et al., 2018; Ma et al., 2019). Besides, the percentage of apoptotic calvarial osteoblast cells derived from Lrp5-knockout mice was significantly decreased after LiCl treatment (Clement-Lacroix et al., 2005).

### Effects of Lithium on Osteoclastogenesis

Osteoclasts are highly specialized cells derived from the monocyte or macrophage lineage of the bone marrow (Tevlin et al., 2014). Several groups of researchers investigated the effects of lithium on osteoclastogenesis using bone marrow macrophages induced with M-CSF and RANKL. The bone resorption pit area, Oc.N, and TRAP activity were decreased in response to LiCl (Hu et al., 2017; Bai et al., 2019). Similarly, the number of osteoclasts was also reduced in bone marrow macrophages seeded on Li-SLA coated surface (Huang et al., 2019). Using murine osteoclast precursor (RAW-D) cells incubated with RANKL, LiCl significantly decreased the number of TRAP-positive multinucleated cells and TRAP activity (Arioka et al., 2014).

In an earlier study by Pepersack et al. (1994), the researchers found that lithium at the dose of 3 mM exerted different effects on resorption stimulated by PTH, prostaglandin E_2_ (PGE_2_), interleukin (IL)-1, or 1,25-dihydroxyvitamin D_3_ [1,25(OH)_2_D_3_] in foetal long bones. Lithium exhibited no inhibitory effect on resorption stimulated by IL-1, followed by slight and moderate inhibitory effects on resorption by PTH and PGE_2_, respectively. Meanwhile, complete inhibition of resorption induced by 1,25(OH)_2_D_3_ after addition of lithium to the bone cultures. The distinct outcomes observed in this study compared to other recent studies might be due to the different approach used for the determination of bone resorption, i.e., measurement of the percentage of calcium release from foetal rat long bones (Pepersack et al., 1994).

Collectively, the potential positive outcomes of lithium observed in preclinical settings prompt a need to look into the underlying molecular action of lithium.

### The Canonical Wingless (Wnt) Signaling Pathway

The canonical Wnt signal transduction pathway has been identified for its central regulatory role in bone metabolism (Houschyar et al., 2019). Activation of this signaling pathway results in the translocation of β-catenin into the nucleus for transcription of genes that belong to the T-cell factor (TCF)/lymphoid enhancer-binding factor (LEF) family. This is an osteogenic signaling cascade which is associated with the upregulation of Runx-2 expression, an essential transcription factor for osteoblast differentiation (Ling et al., 2017). In the presence of Wnt ligand, it binds to transmembrane Frizzled receptor and Lrp5/6 co-receptors. The function of multiprotein destruction complex made of axis inhibition protein 2 (Axin2), adenomatosis polyposis coli (APC), GSK3β, and casein kinase 1 alpha (CK1α) is disrupted. Eventually, the degradation of β-catenin is inhibited. The accumulation of β-catenin in cytoplasm facilitates its translocation and subsequent gene transcription. In the case whereby Wnt ligand is absent, β-catenin is sequestered into the multiprotein destruction complex, phosphorylated, ubiquitinylated, followed by the degradation by the proteasome (Houschyar et al., 2019).

In particular, the activity of Wnt signaling is constitutively suppressed by the active form of GSK3β under basal condition (Muneer, 2017). The inactivation (phosphorylation) of GSK3β leads to dephosphorylation of β-catenin and activation of Wnt signaling to promote bone anabolism (Bertacchini et al., 2018). This pathway has been regarded as the most implicated signaling mechanism in the regulation of osteoblastogenesis and bone formation by lithium due to its well-established anti-GSK3β property (Kim et al., 2013). A study by Arioka et al. (2014) examined whether GSK3β inhibitors (LiCl and SB216763) were effective in modulating osteoblastogenesis of mesenchymal progenitor C3H10T1/2 cells. Both inhibitors promoted osteoblast differentiation and caused dephosphorylation of β-catenin (Arioka et al., 2014). For bone-repairing effects, Li et al. (2018) fabricated a composited scaffold using lithium (Li-nHA) to activate Wnt signaling pathway and consequently improve osteogenic effects in bone marrow MSCs. The co-cultures of bone marrow MSCs and Li-nHA had lower expression of GSK3β and peroxisome proliferator-activated receptor-gamma (PPAR-γ) but higher expression of β-catenin (Li et al., 2018). Likewise, Western analysis revealed that β-catenin was upregulated in bone marrow MSCs seeded on titanium discs coated with Li-SLA (Huang et al., 2019). Another group of investigators also conducted *in vitro* experiment to examine the fracture-healing effects of lithium-doped CPC on MC3T3-E1 cells. The release of lithium ions from CPC increased the amount of phosphorylated GSK3β but decreased the amount of phosphorylated β-catenin (Li et al., 2017a). A more comprehensive study was performed to investigate the involvement of Wnt signaling. Human osteosarcoma (MG-63) cells were seeded on Li-MAO-ETP scaffold and the expression of all signaling molecules downstream the Wnt signaling was evaluated. The expressions of Lrp5, Lrp6, Axin2, β-catenin, and phosphorylated GSK3β were increased whereas the expression of phosphorylated β-catenin was decreased (Liu et al., 2018). A recent study showed similar outcomes when human osteosarcoma (MG-63) cells were cultured on Li-CPP. Cell immunofluorescence staining and Western analysis showed lower expression of GSK3β but higher expression of β-catenin. Higher content of lithium caused a further decrease in GSK3β expression and increase in β-catenin expression (Ma et al., 2019). Mechanistically, LiCl inhibited GSK3β and caused the accumulation of β-catenin in cytoplasm leading to its translocation into the nucleus and expression of Runx-2 (one of the TCF/LEF target gene products essential for osteoblast differentiation) (Arioka et al., 2014; Li et al., 2017a; Li et al., 2018; Liu et al., 2018; Ma et al., 2019).

Clement-Lacroix et al. (2005) provided strong evidence on the activation of Wnt signaling by LiCl in increasing bone formation and bone mass using Lrp5-knockout mice. The lack of Lrp5 in mice caused the failure of Wnt signaling *via* the canonical pathway, leading to reduced bone mass. LiCl was predicted to act downstream of this receptor to express Wnt-responsive genes and restore bone mass. *In vitro*, LiCl was able to increase β-catenin stabilization and induce β-catenin nuclear translocation in calvaria cells derived from Lrp5-knockout mice. The ALP activity and COL1α1 expression were also stimulated in MSCs obtained from Lrp5-knockout mice (Clement-Lacroix et al., 2005). The study of Kurgan et al. (2019) evaluated the effects of LiCl treatment (10 mg/kg) on GSK3β phosphorylation status within the bone in male C57BL/6J mice. They pointed out that lithium supplementation caused phosphorylation of GSK3β, rendering it inactive and dephosphorylation of β-catenin (Kurgan et al., 2019). Other important players in Wnt signaling include Dickkopf-related protein 1 (DKK-1) and sclerostin (SOST). They are osteocyte-derived Wnt antagonist function as putative inhibitors of Wnt signaling to block the engagement of Wnt ligand to Frizzled receptor and Lrp5/6 co-partners (Mason and Williams, 2010). In a fractured mouse model, researchers demonstrated that the expression of β-catenin was attenuated whereas the expression of SOST and GSK3β were enhanced along with the impairments in fracture healing. These alterations in fracture healing in mice were rescued by administering LiCl (Loiselle et al., 2013).

The role of Wnt signaling in the regulation of osteoblastogenesis has been generally accepted but the involvement of this pathway in osteoclastogenesis remains controversial based on several lines of evidence. The inactivation of GSK3β by RANKL is crucial for osteoclast differentiation, confirming the negative role of GSK3β in osteoclast formation (Jang et al., 2011). On the contrary, some studies reported that constitutive activation of β-catenin prevents osteoclast differentiation (Wei et al., 2011; Albers et al., 2013). The exact role of Wnt signaling in osteoclastogenesis awaits further investigation. The effects of lithium on osteoclastogenesis did not seem to mediate through Wnt signaling. A study by Arioka et al. (2014) examined whether LiCl and SB216763 were effective in modulating osteoclastogenesis of osteoclast precursor RAW-D cells in the presence of RANKL. Their findings revealed that GSK3β inhibitors suppressed osteoclast differentiation [assessed by reduced TRAP activity, number of multinucleated cells and nuclear factor of activated T-cells cytoplasmic 1 (NFATc1) expression] independently of Wnt pathway. This was mainly because Wnt3a stimulated osteoclast formation from their precursor RAW-D cells (Arioka et al., 2014). These findings were supported by another study whereby LiCl-treated bone marrow macrophages in the presence of M-CSF and RANKL displayed a reduction in osteoclast formation but treatment with a selective inhibitor of GSK3β (IM-12) showed vice versa (Hu et al., 2017). Taken together, these studies suggested that LiCl-induced inhibition of osteoclastogenesis was not affected by the Wnt signaling pathway.

### The Phosphatidylinositol 3-Kinase (PI3K)/Protein Kinase B (Akt) Signaling Pathway

The PI3K/Akt signaling pathway is a pathway essential for cell survival, growth and proliferation (Shi et al., 2019). This pathway is activated by the binding of ligands (such as hormones, growth factors and extracellular matrix components) with receptor tyrosine kinase (RTK) resulting in dimerization and autophosphorylation of tyrosine residues in the cytoplasmic domain. The phosphorylated tyrosine residues recruit PI3K (consists of a regulatory p85 subunit and a catalytic p110 subunit) to convert phosphatidylinositol-4,5-diphosphate (PIP_2_) into phosphatidylinositol-3,4,5-triphosphate (PIP_3_). Next, PIP_3_ acts as a secondary messenger to recruit and bind to phosphoinositide-dependent kinase-1 (PDK1) and Akt. Upon binding, PDK1 phosphorylates Akt causing full activation of Akt (McGonnell et al., 2012). GSK3β is a major effector of Akt and NFATc1 is a direct substrate of GSK3β, thus activated Akt subsequently inhibits GSK3β and downregulates NFATc1 expression (Moon et al., 2012).

Bai et al. (2019) examined the role of lithium on Akt/GSK3β/β-catenin/NFATc1 signaling pathway in osteoblasts and osteoclasts. They harvested primary osteoblasts from the calvariae of 3-day-old rats and generated osteoclasts using bone marrow-derived macrophages in the presence of M-CSF and RANKL. Both osteoblasts and osteoclasts were administered with LiCl and LY294002 (a known PI3K inhibitor) alone or in combination. Treatment with LiCl or LY294002 alone caused phosphorylation of Akt and GSK3β but dephosphorylation of β-catenin in osteoblasts and osteoclasts along with other findings (the increases in ALP activity and mineralized nodules in osteoblast as well as reductions of the resorption pit area, TRAP activity and NFATc1 expression). The trend became more evident when the combination of LiCl and LY294002 was administered (Bai et al., 2019). Li and colleagues also demonstrated the role of Li-nHA in the differentiation of MSCs into osteoblasts *via* PI3K/Akt signaling pathway. Their mechanistic study showed that Li-nHA scaffold co-cultured with bone marrow MSCs had more Akt expression as compared to the negative control group (Li et al., 2018). The activation of PI3K/Akt pathway has direct and indirect effects on osteogenic stimulation. The indirect effect is mediated *via* intersection with canonical Wnt signaling.

### The Bone Morphogenetic Protein-2 (BMP-2) Signaling Pathway

Bone morphogenetic proteins (BMPs), belong to transforming growth factor-beta (TGF-β) gene superfamily, are found abundantly in bone and cartilage thus important for skeletal development and tissue regeneration (Wu et al., 2016). They signal through binding with tetrameric transmembrane serine/threonine kinase receptor (BMPR) expressed on the surface of osteoblast and osteoclast precursors. The downstream intracellular signaling events are mediated through canonical suppressor of mothers against decapentaplegic (SMAD)-dependent and non-canonical SMAD-independent pathways, such as mitogen-activated protein kinase (MAPK) signaling cascade. Both of these signal transduction pathways direct the transcription of osteogenic genes (Wu et al., 2016).

The findings from studies by two groups of researchers suggested that lithium inhibited osteogenic commitment of MSCs but enhanced osteogenic differentiation at the later stage, whereby both actions were mediated *via* BMP-2 signaling (Li et al., 2011; Huang et al., 2019). In an earlier study by Li and colleagues, LiCl attenuated BMP-2 signaling and inhibited early stages of osteogenic differentiation. The presence of LiCl reduced mineral deposition in von Kossa staining and ALP activity in MC3T3-E1 pre-osteoblast cultures as early as day 2. The expression of osteoblast-specific transcription factors (*Runx-2*, *OSX*, *Atf4*, *Dlx5*, *Alpl*, *Col1α1*, *Ibsp*, *Spp1*, and *OCN*) in mouse primary osteoblasts was also reduced at day 3 but these inhibitory effects of LiCl were diminished at day 6 and completely abolished at day 9. Comparably, lithium treatment also downregulated osteogenic markers (*Runx-2*, *OSX*, *Alpl*, and *OCN*) in murine pluripotent mesenchymal (C2C12) cells (Li et al., 2011). In a recent study, it was found that bone marrow MSCs incubated on Li-SLA coated titanium discs displayed upregulation of BMP-2 after 7 days (Huang et al., 2019).

### The Receptor Activator of Nuclear Factor-Kappa B (RANK)/Receptor Activator of Nuclear Factor-Kappa B (RANKL)/Osteoprotegerin (OPG) System

The RANK/RANKL/OPG signaling axis, identified in the mid-to-late 1990s, shows an essential role of osteoblasts in modulating osteoclast formation and differentiation (Boyce and Xing, 2007). The binding of RANKL to its receptor, RANK on osteoclast precursors or osteoclasts initiates signals mediated by tumour necrosis factor receptor-associated factor 6 (TRAF6) adaptor protein and promptly causes activation of various downstream signaling events, including NF-κB, MAPK, PI3K/Akt, and NFATc1 signaling cascades (Boyle et al., 2003; Lee and Kim, 2003; Luo et al., 2016). OPG is a protein secreted by osteoblasts and it acts as a decoy receptor by binding with RANKL to negatively regulate the RANK/RANKL/OPG system thus suppressing the formation, fusion, activity, and survival of osteoclasts. Several groups of researchers delineated the differential expression of OPG and RANKL (characterized by raised OPG and unchanged RANKL) in murine mesenchymal and calvaria cells after plated on Li-SLA-coated titanium discs (Galli et al., 2013; Huang et al., 2019). A similar trend was observed, whereby there was an increase in OPG but RANKL level was unaltered in the femur of male C57BL/6J mice after treated with LiCl (10 mg/kg) (Kurgan et al., 2019). Furthermore, lithium was shown to suppress TRAP activity and TRAP-positive cell, indicating the reduction of osteoclast formation in osteoclast precursor cells cultured in the presence of M-CSF and RANKL (Arioka et al., 2014; Hu et al., 2017; Bai et al., 2019; Huang et al., 2019).

NFATc1 has been characterized as a master regulator during osteoclastogenesis and it can be induced in three different stages of osteoclastogenesis (Park et al., 2017). In the early stage, the activated NF-κB in the RANK/RANKL/OPG system induces the expression of NFATc1. In the intermediate stage, NFATc1 can be further activated *via* calcium signaling. The cooperation between RANKL-RANK signaling and co-stimulatory receptors [triggering receptors expressed on myeloid cells 2 (TREM-2)/DNAX-activating protein of 12 kDa (DAP12) and osteoclast-associated receptor (Oscar)/Fc receptor gamma (FcRγ)] activate phospholipase Cγ2 (PLCγ2) to facilitate robust amplification of NFATc1. At the late stage of osteoclastogenesis, NFATc1 translocates into the nucleus to induce osteoclast-specific target genes for osteoclast fusion and function (Park et al., 2017). The effects of LiCl on the expression of NFATc1 were also assessed. Bone marrow macrophages stimulated by M-CSF and RANKL expressed higher levels of NFATc1 and the expression was suppressed by LiCl in dose-dependent and time-dependent manner (Bai et al., 2019).

### The Regulation of Inflammatory Response *via* Nuclear Factor-Kappa B (NF-κB) and Mitogen-Activated Protein Kinase (MAPK) Signaling Pathways

The NF-κB and MAPK signaling play a significant role in inflammation. NF-κB represents a set of inducible transcription factors regulating the production of cytokines involved in immune and inflammatory responses (Liu et al., 2017). NF-κB, which exists in dimers, either homodimers or heterodimers, is typically sequestered in the cytoplasm by the inhibitor of NF-κB (IκBα). NF-κB can be activated by many influencers, including the interaction between RANKL, tumour necrosis factor-alpha (TNF-α) and IL-1 with their respective receptors. Eventually, NF-κB modulates the synthesis of cytokines producing a self-amplifying cycle of cytokine release and NF-κB activation (Boyce et al., 2010). MAPKs are highly conserved protein kinases that phosphorylate their dual serine and threonine residues, also known as autophosphorylation (Soares-Silva et al., 2016). The MAPK signaling can be activated in response to pro-inflammatory stimuli, leading to activation of transcription factors such as activator protein (AP-1) and subsequent expression of inflammatory genes (Yang et al., 2014). The overwhelming inflammatory response stimulated by NF-κB and MAPK activation is closely associated with perturbation of bone metabolism, hence promoting the initiation and progression of osteoporosis and other bone-related disorders.

Hu and co-authors recently reported that LiCl reduced the levels of TNF-α, IL-1β, and IL-6 with improvement in bone microstructure in a mouse model of calvarial osteolysis, indicating that LiCl effectively alleviated inflammatory response and bone loss. Their concurrent *in vitro* study showed that bone marrow macrophages treated with M-CSF, RANKL, and exposed to LiCl had lower osteoclast formation and osteoclast-related gene expression [such as NFATc1, CTSK, semaphorin-4A (Sema-4A) and Oscar]. The inhibitory effects of LiCl in osteoclastogenesis were mainly regulated *via* the inhibition of NF-κB. The phosphorylation of IκBα and NF-κB p65, as well as translocation of p65 proteins from cytoplasm to nucleus, occurred upon stimulation with RANKL alone. These changes were diminished by LiCl treatment. LiCl also suppressed the protein expression of Fos proto-oncogene (c-Fos) and NFATc1, which are the transcription factors to promote osteoclast differentiation. For other signaling pathways downstream the interaction between RANKL and RANK, Western analysis revealed that none of the subfamilies of MAPK [including the extracellular-signal-regulated kinase (ERK), c-Jun N-terminal kinase (JNK) and p38 MAPK] and PI3K/Akt was affected by LiCl (Hu et al., 2017). In a recent study, Yang et al. (2019) showed that LiCl reduced titanium nanoparticle-stimulated inflammatory response in the RAW264.7 macrophages. Specifically, titanium particle stimulation increased production of pro-inflammatory (TNF-α and IL-6) but reduced secretion of anti-inflammatory cytokines (IL-4 and IL-10). These changes were reversed in the presence of LiCl. Western blot results also revealed that titanium particle stimulation significantly increased ERK and p38 MAPK phosphorylation. The activation of these signaling events was suppressed by LiCl treatment (Yang et al., 2019).

**Figure 1:**
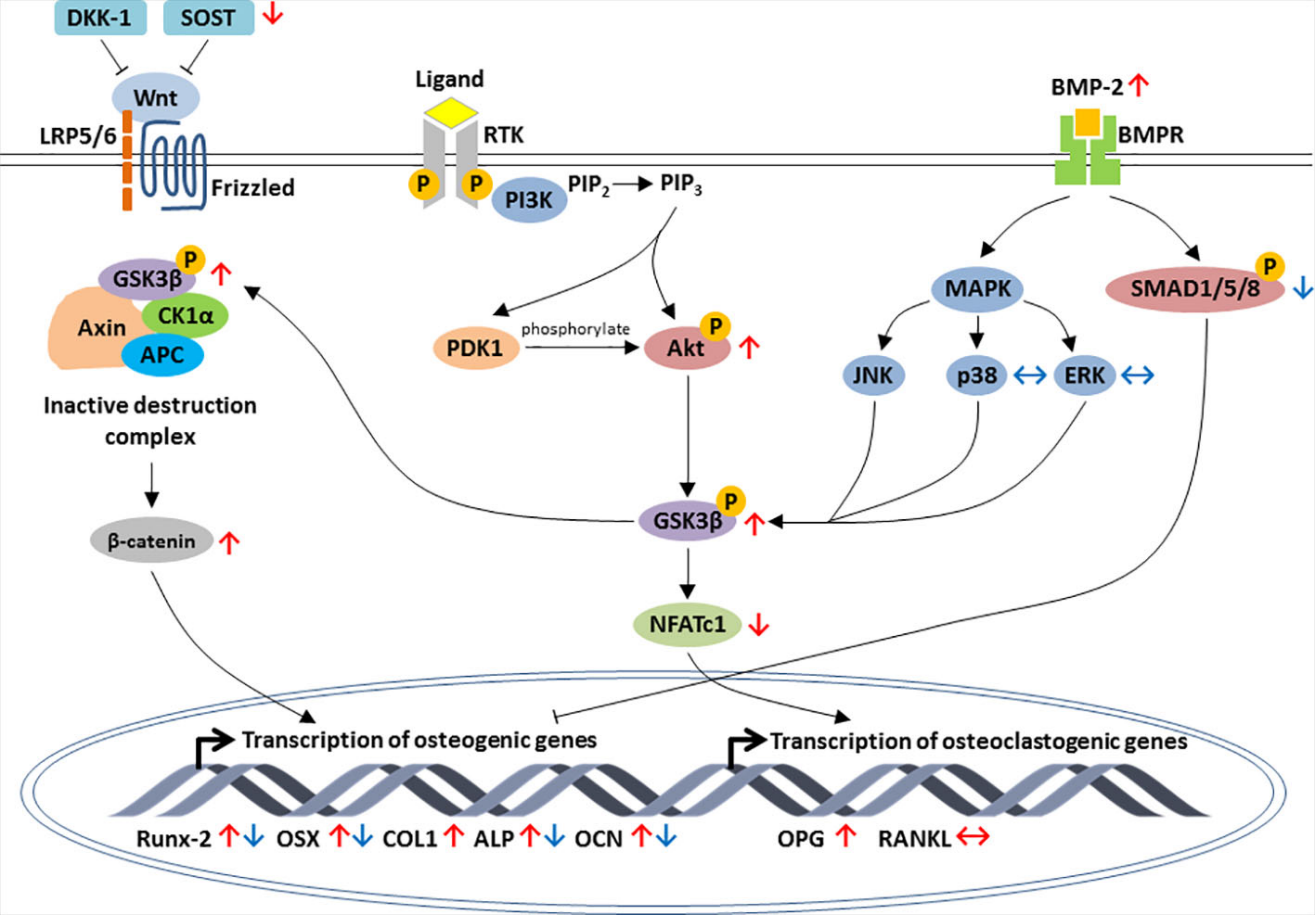
Schematic diagram depicting the regulation of osteoblastic-specific genes expression by lithium through activation of three major signaling pathways, including the canonical Wnt/β-catenin, PI3K/Akt and BMP-2 signaling pathways. In Wnt/β-catenin pathway, lithium inhibits the expression of sclerostin (SOST) as well as increases phosphorylation of GSK3β and accumulation of β-catenin. Through PI3K/Akt pathway, the activation of Akt by lithium leads to the inhibition of GSK3β and downregulation of NFATc1. Lithium also enhances osteogenic differentiation by increasing BMP-2 expression. The effects of lithium are indicated by red arrows. For early stage of osteogenic differentiation, lithium inhibited the expression of osteogenic markers by increasing phosphorylation of suppressor of mothers against decapentaplegics (SMADs) in BMP-2 signal transduction pathway (the effects of lithium are indicated by blue arrows). Arrow pointing upward (↑) indicates an increase and pointing downward (↓) indicates a decrease. Equivalent arrow (↔) indicates no effect.

**Figure 2:**
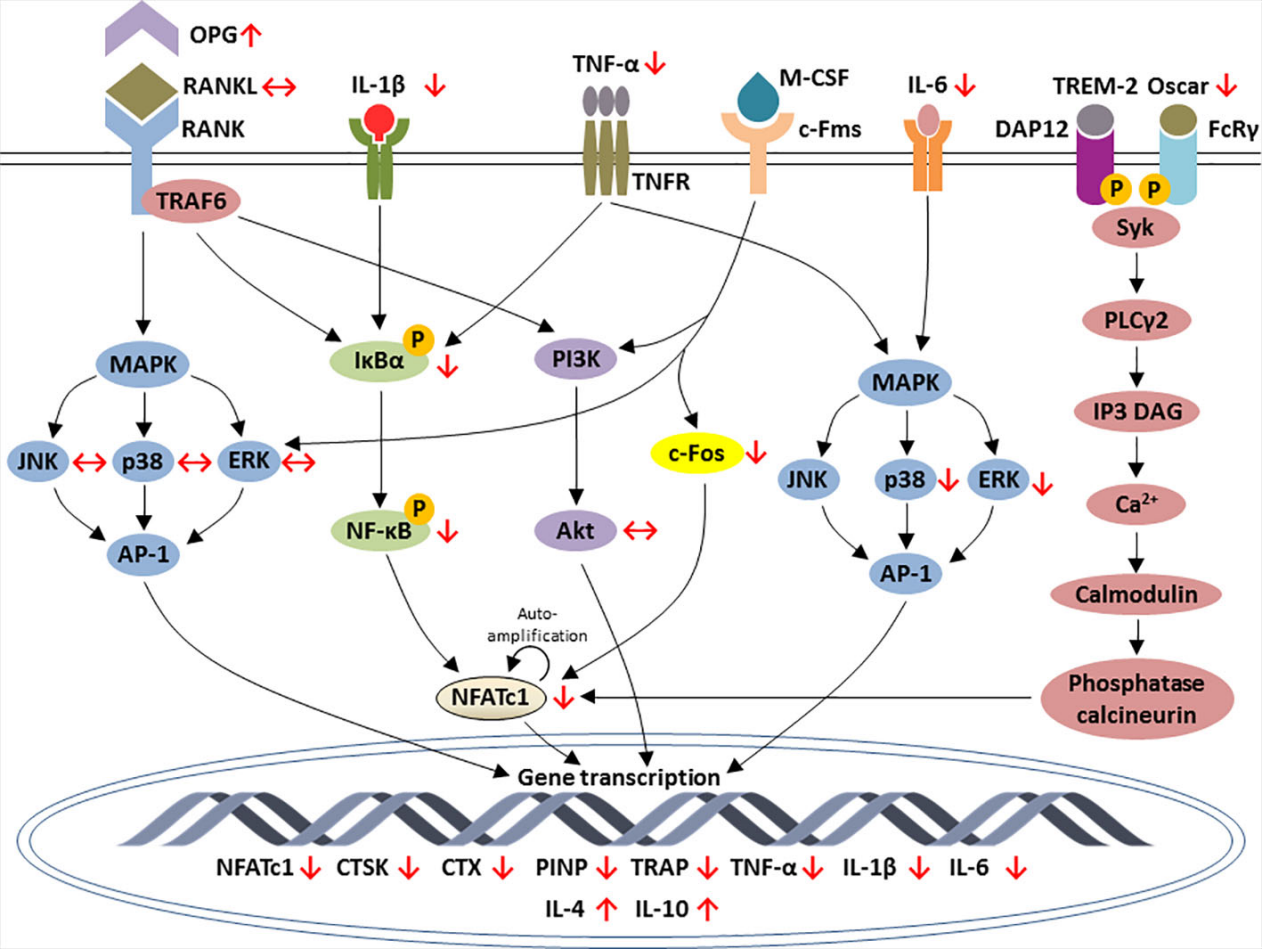
Schematic diagram depicting the regulation of osteoclastic-specific genes expression by lithium through macrophage-colony stimulating factor (M-CSF), nuclear factor-kappa B (RANK)/RANK ligand (RANKL)/osteoprotegerin (OPG), NF-κB, mitogen-activated protein kinase (MAPK), and calcium signaling pathways. Lithium increases the expression of OPG and reduces the inflammatory response that eventually leads to the inhibition of NF-κB and NFATc1. Lithium downregulates c-Fos, a downstream target of interaction between M-CSF and c-Fms, which is essential for osteoclast differentiation. Lithium exerts differential outcomes for MAPK signaling. The increases in TNF-α and IL-6 by lithium subsequently inhibits ERK and p38 phosphorylation but none of the subfamilies of MAPK is affected downstream the inhibition of RANK-RANKL interaction. In calcium signaling pathways, lithium inhibits Oscar which an important bone-specific regulator for osteoclast differentiation *via* NFATc1 amplification. The effects of lithium are indicated by red arrows. Arrow pointing upward (↑) indicates an increase and pointing downward (↓) indicates a decrease. Equivalent arrow (↔) indicates no effect.

To sum up, most of these animal and cell culture studies pointed out that lithium promoted osteoblastogenesis and bone formation but inhibited osteoclastogenesis and bone resorption. The modulation of osteogenesis is mainly through activation of Wnt, PI3K/Akt and BMP-2 signaling axis (**Figure 1**). The inhibition of osteoclastogenesis by lithium is orchestrated through the RANK/RANKL/OPG system and the regulation of inflammatory response *via* NF-κB and MAPK signaling pathways (**Figure 2**).

## Challenge for the Use of Lithium

The major medical challenge of lithium is the underutilization of clinical application mainly attributable to the presence of unfavourable side effects (Rybakowski, 2018), despite the reported favourable effects and the necessity of trace amount of lithium (1000 mg/day for a 70 kg adult) for mental and physical health (Schrauzer, 2002). Long-term lithium therapy potentially comes at the cost of renal, endocrine and dermatological side effects. In the kidney, 4 weeks of lithium therapy caused renal tubular concentration defects (characterized by a reduction in urinary concentrating ability) in healthy volunteers with greater concern of developing polyuria, polydipsia, and nephrogenic diabetes insipidus (Walker et al., 2005). Lithium treatment was also closely associated with hyperparathyroidism and hypercalcaemia. A case report revealed that a 28-year-old female patient who receiving Li_2_CO_3_ for bipolar mood disorder for at least 2 years developed hyperparathyroidism (Nair et al., 2013). In a cohort study, it was found that bipolar patients with lithium treatment had higher odds of hypercalcaemia as compared to those not receiving lithium treatment (Meehan et al., 2018). Otherwise, the skin is adversely affected by lithium. A case report reported that a 31-year-old Caucasian woman with bipolar disorder type I manifested a cutaneous adverse reaction after treated with Li_2_CO_3_ (Pinna et al., 2017). Some clinical cohort studies also indicated the association between lithium treatment and congenital malformation, raising the concern about the possible teratogenicity of lithium exposure during the first trimester (Poels et al., 2018).

## Research Gap and Future Perspectives

The efficacy of lithium in promoting bone health has been abundantly demonstrated by researchers in controlled experimental settings and human populations. This collection of articles encourages the greater use of lithium despite as a fundamental treatment for bipolar disease. To our knowledge, other commonly used medications for bipolar disease such as anti-depressants, anti-convulsants, and neuroleptics are associated with low BMD (Crews and Howes, 2012; Rady et al., 2018; Zhou et al., 2018). Lithium may be a better treatment option for bipolar disease patients with low BMD taking into consideration the side effects of lithium. Hence, the clinical utility of lithium particularly the dose and frequency of supplementation should be under careful consideration to maximize its efficacy and prevent life-threatening complications.

Lithium targets the perturbation of canonical Wnt/β-catenin, PI3K/Akt, BMP-2, RANKL/RANKL/OPG, NF-κB, MAPK, and calcium signaling responsible for bone pathologies. It becomes clear that GSK3β may be a central nexus to cross-talk in many signaling pathways and transcriptional networks controlling bone cell development to fine-tune the bone remodelling processes. The current state of knowledge suggests that GSK3β may be a potential drug target for the management of osteoporosis, fractures and bone defects. However, GSK3β is a multifaceted protein kinase involved in the regulation of many cellular activities including energy metabolism and signaling by cytokines and hormones. The inhibition of GSK3 by lithium may suggest the multiple adverse effects of lithium. Apart from suggesting lithium as a bone-protective agent and understanding the molecular action of lithium on bone, this review provides new insights into repurposing other GSK3β inhibitors as a potential adjunct therapy for bone-related conditions.

## Conclusion

It is tempting to speculate lithium as a promising pharmaceutical agent toward improving bone health. Apart from exerting its beneficial properties on bone *via* direct administration, the application of lithium in bone tissue engineering has been attempted by doping a trace amount on the scaffold to enhance osteogenesis and inhibit osteoclastogenesis subsequently improve the physical-mechanical characteristics of bone. In term of treating bone defect and fracture, lithium seems to be a good choice of microelement for improving the shortcomings of synthetic bone substitutes for their brittleness and insufficiency to promote osteogenesis. Lithium is also applicable for bipolar disease patients with the phenotype of low bone mass who are prone to osteoporosis-related fractures. We hope this review may provide a small contribution to bring novel possibilities for the additional therapeutic use of lithium, particularly against bone-related disorders, in patients with psychiatric problems. A comprehensive understanding of the mechanism of action for lithium in bone metabolism is likely to reveal the potential molecular targets for novel bone therapies.

## Author Contributions

SW performed literature search and drafted the manuscript. K-YC and SI-N provided critical review for the manuscript. SI-N gave the final approval for the publication of this manuscript.

## Funding

We thank Universiti Kebangsaan Malaysia and Ministry of Education, Malaysia for supporting this work *via* MI-2019-006 and FRGS/1/2018/SKK10/UKM/03/1 grants.

## Conflict of Interest

The authors declare that the research was conducted in the absence of any commercial or financial relationships that could be construed as a potential conflict of interest.

## References

[B1] Albers J. Keller J. Baranowsky A. Beil F. T. Catala-Lehnen P. Schulze J. (2013). Canonical Wnt signaling inhibits osteoclastogenesis independent of osteoprotegerin. J. Cell Biol. 200 (4), 537–549. 10.1083/jcb.201207142 23401003 PMC3575535

[B2] Arioka M. Takahashi-Yanaga F. Sasaki M. Yoshihara T. Morimoto S. Hirata M. (2014). Acceleration of bone regeneration by local application of lithium: Wnt signal-mediated osteoblastogenesis and Wnt signal-independent suppression of osteoclastogenesis. Biochem. Pharmacol. 90 (4), 397–405. 10.1016/j.bcp.2014.06.011 24955980

[B3] Bai J. Xu Y. Dieo Y. Sun G. (2019). Combined low-dose LiCl and LY294002 for the treatment of osteoporosis in ovariectomized rats. J. Orthop. Surg. Res. 14 (1), 177. 10.1186/s13018-019-1210-1 31196133 PMC6567919

[B4] Baran D. T. Schwartz M. P. Bergfeld M. A. Teitelbaum S. L. Slatopolsky E. Avioli L. V. (1978). Lithium inhibition of bone mineralization and osteoid formation. J. Clin. Invest. 61 (6), 1691–1696. 10.1172/JCI109090 659622 PMC372696

[B5] Bellwinkel S. Schafer A. Minne H. Ziegler R. (1975). The effect of chronic lithium application on the mineral content of rat bone and soft tissues. Int. Pharmacopsychiatry 10 (1), 9–16. 10.1159/000468163 1140905

[B6] Bernick J. Wang Y. Sigal I. A. Alman B. A. Whyne C. M. Nam D. (2014). Parameters for lithium treatment are critical in its enhancement of fracture-healing in rodents. J. Bone Joint Surg. Am. 96 (23), 1990–1998. 10.2106/JBJS.N.00057 25471914 PMC4249593

[B7] Bertacchini J. Magarò M. S. Potì F. Palumbo C. (2018). Osteocytes Specific GSK3 Inhibition Affects In Vitro Osteogenic Differentiation. Biomedicines 6 (2), 61. 10.3390/biomedicines6020061 PMC6027076 29883388

[B8] Bolton J. M. Metge C. Lix L. Prior H. Sareen J. Leslie W. D. (2008). Fracture risk from psychotropic medications: a population-based analysis. J. Clin. Psychopharmacol. 28 (4), 384–391. 10.1097/JCP.0b013e31817d5943 18626264

[B9] Bolton J. M. Morin S. N. Majumdar S. R. Sareen J. Lix L. M. Johansson H. (2017). Association of Mental Disorders and Related Medication Use With Risk for Major Osteoporotic Fractures. JAMA Psychiatry 74 (6), 641–648. 10.1001/jamapsychiatry.2017.0449 28423154 PMC5539842

[B10] Bonjour J.-P. Chevalley T. Ferrari S. Rizzoli R. (2009). The importance and relevance of peak bone mass in the prevalence of osteoporosis. Salud Publica Mex. 51 (Suppl 1), S5–S17. 10.1590/S0036-36342009000700004 19287894

[B11] Boyce B. F. Xing L. (2007). The RANKL/RANK/OPG pathway. Curr. Osteoporos Rep. 5 (3), 98–104. 10.1007/s11914-007-0024-y 17925190

[B12] Boyce B. F. Yao Z. Xing L. (2009). Osteoclasts have multiple roles in bone in addition to bone resorption. Crit. Rev. Eukaryot Gene Expr. 19 (3), 171–180. 10.1615/CritRevEukarGeneExpr.v19.i3.10 19883363 PMC2856465

[B13] Boyce B. F. Yao Z. Xing L. (2010). Functions of nuclear factor kappaB in bone. Ann. N. Y. Acad. Sci. 1192, 367–375. 10.1111/j.1749-6632.2009.05315.x 20392262 PMC3013362

[B14] Boyle W. J. Simonet W. S. Lacey D. L. (2003). Osteoclast differentiation and activation. Nature 423 (6937), 337–342. 10.1038/nature01658 12748652

[B15] Chapurlat R. D. Confavreux C. B. (2016). Novel biological markers of bone: from bone metabolism to bone physiology. Rheumatology 55 (10), 1714–1725. 10.1093/rheumatology/kev410 26790456

[B16] Clement-Lacroix P. Ai M. Morvan F. Roman-Roman S. Vayssiere B. Belleville C. (2005). Lrp5-independent activation of Wnt signaling by lithium chloride increases bone formation and bone mass in mice. Proc. Natl. Acad. Sci. U. S. A 102 (48), 17406–17411. 10.1073/pnas.0505259102 16293698 PMC1297659

[B17] Cohen O. Rais T. Lepkifker E. Vered I. (1998). Lithium carbonate therapy is not a risk factor for osteoporosis. Horm. Metab. Res. 30 (9), 594–597. 10.1055/s-2007-978939 9808330

[B18] Crews M. P. K. Howes O. D. (2012). Is antipsychotic treatment linked to low bone mineral density and osteoporosis? A review of the evidence and the clinical implications. Hum. Psychopharmacol. 27 (1), 15–23. 10.1002/hup.1265 22228316 PMC3731625

[B19] Dai F. Zhang F. Sun D. Zhang Z. H. Dong S. W. Xu J. Z. (2015). CTLA4 enhances the osteogenic differentiation of allogeneic human mesenchymal stem cells in a model of immune activation. Braz. J. Med. Biol. Res. 48 (7), 629–636. 10.1590/1414-431x20154209 26017342 PMC4512102

[B20] Davis J. M. Janicak P. G. Hogan D. M. (1999). Mood stabilizers in the prevention of recurrent affective disorders: a meta-analysis. Acta Psychiatr. Scand. 100 (6), 406–417. 10.1111/j.1600-0447.1999.tb10890.x 10626918

[B21] Eldar-Finkelman H. Martinez A. (2011). GSK-3 Inhibitors: Preclinical and Clinical Focus on CNS. Front. Mol. Neurosci. 4, 32–32. 10.3389/fnmol.2011.00032 22065134 PMC3204427

[B22] Embi N. Rylatt D. B. Cohen P. (1980). Glycogen synthase kinase-3 from rabbit skeletal muscle. Separation from cyclic-AMP-dependent protein kinase and phosphorylase kinase. Eur. J. Biochem. 107 (2), 519–527. 10.1111/j.1432-1033.1980.tb06059.x 6249596

[B23] Eren I. Yildiz M. Civi I. (2006). The effects of lithium treatment on bone mineral density in bipolar patients. Neurol. Psychiat Br. 13 (4), 174–179.

[B24] Forlenza O. V. De-Paula V. J. R. Diniz B. S. O. (2014). Neuroprotective effects of lithium: implications for the treatment of Alzheimer’s disease and related neurodegenerative disorders. ACS Chem. Neurosci. 5 (6), 443–450. 10.1021/cn5000309 24766396 PMC4063497

[B25] Galli C. Piemontese M. Lumetti S. Manfredi E. Macaluso G. M. Passeri G. (2013). GSK3b-inhibitor lithium chloride enhances activation of Wnt canonical signaling and osteoblast differentiation on hydrophilic titanium surfaces. Clin. Implants Res. 24 (8), 921–927. 10.1111/j.1600-0501.2012.02488.x 22626030

[B26] Gelenberg A. J. Hopkins H. S. (1993). Report on efficacy of treatments for bipolar disorder. Psychopharmacol. Bull. 29 (4), 447–456. 8084977

[B27] Guo H. Wang C. Wang J. He Y. (2018). Lithium-incorporated deproteinized bovine bone substitute improves osteogenesis in critical-sized bone defect repair. J. Biomater. Appl. 32 (10), 1421–1434. 10.1177/0885328218768185 29703129

[B28] Harvey B. M. Eschbach M. Glynn E. A. Kotha S. Darre M. Adams D. J. (2015). Effect of daily lithium chloride administration on bone mass and strength in growing broiler chickens. Poult. Sci. 94 (2), 296–301. 10.3382/ps/peu079 25609690

[B29] Henneman D. Zimmerberg J. J. (1974). Lack of effect of chronic lithium chloride on bone composition and metabolism. Endocrinology 94 (3), 915–917. 10.1210/endo-94-3-915 4813687

[B30] Houschyar K. S. Tapking C. Borrelli M. R. Popp D. Duscher D. Maan Z. N. (2019). Wnt Pathway in Bone Repair and Regeneration - What Do We Know So Far. Front. Cell Dev. Biol. 6, 170–170. 10.3389/fcell.2018.00170 30666305 PMC6330281

[B31] Hu X. Wang Z. Shi J. Guo X. Wang L. Ping Z. (2017). Lithium chloride inhibits titanium particle-induced osteoclastogenesis by inhibiting the NF-kappaB pathway. Oncotarget 8 (48), 83949–83961. 10.18632/oncotarget.20000 29137395 PMC5663567

[B32] Huang T. B. Li Y. Z. Yu K. Yu Z. Wang Y. Jiang Z. W. (2019). Effect of the Wnt signal-RANKL/OPG axis on the enhanced osteogenic integration of a lithium incorporated surface. Biomater. Sci. 7 (3), 1101–1116. 10.1039/C8BM01411F 30633253

[B33] Jang H. D. Shin J. H. Park D. R. Hong J. H. Yoon K. Ko R. (2011). Inactivation of glycogen synthase kinase-3beta is required for osteoclast differentiation. J. Biol. Chem. 286 (45), 39043–39050. 10.1074/jbc.M111.256768 21949120 PMC3234729

[B34] Jin Y. Xu L. Hu X. Liao S. Pathak J. L. Liu J. (2017). Lithium chloride enhances bone regeneration and implant osseointegration in osteoporotic conditions. J. Bone Miner. Metab. 35 (5), 497–503. 10.1007/s00774-016-0783-6 27714461

[B35] Kalu D. N. (1991). The ovariectomized rat model of postmenopausal bone loss. Bone Miner. 15 (3), 175–191. 10.1016/0169-6009(91)90124-I 1773131

[B36] Kim J. H. Liu X. Wang J. Chen X. Zhang H. Kim S. H. (2013). Wnt signaling in bone formation and its therapeutic potential for bone diseases. Ther. Adv. Musculoskelet. Dis. 5 (1), 13–31. 10.1177/1759720X12466608 23514963 PMC3582304

[B37] Kurgan N. Bott K. N. Helmeczi W. E. Roy B. D. Brindle I. D. Klentrou P. (2019). Low dose lithium supplementation activates Wnt/beta-catenin signalling and increases bone OPG/RANKL ratio in mice. Biochem. Biophys. Res. Commun. 511 (2), 394–397. 10.1016/j.bbrc.2019.02.066 30791983

[B38] Lee Z. H. Kim H. H. (2003). Signal transduction by receptor activator of nuclear factor kappa B in osteoclasts. Biochem. Biophys. Res. Commun. 305 (2), 211–214. 10.1016/S0006-291X(03)00695-8 12745060

[B39] Lewicki M. Paez H. Mandalunis P. M. (2006). Effect of lithium carbonate on subchondral bone in sexually mature Wistar rats. Exp. Toxicol. Pathol. 58 (2-3), 197–201. 10.1016/j.etp.2006.06.003 16846729

[B40] Lewitzka U. Severus E. Bauer R. Ritter P. Müller-Oerlinghausen B. Bauer M. (2015). The suicide prevention effect of lithium: more than 20 years of evidence-a narrative review. Int. J. Bipolar Disord. 3 (1), 32–32. 10.1186/s40345-015-0032-2 26183461 PMC4504869

[B43] Li J. Khavandgar Z. Lin S. H. Murshed M. (2011). Lithium chloride attenuates BMP-2 signaling and inhibits osteogenic differentiation through a novel WNT/GSK3- independent mechanism. Bone 48 (2), 321–331. 10.1016/j.bone.2010.09.033 20932949

[B42] Li H. Huang K. Liu X. Liu J. Lu X. Tao K. (2014). Lithium chloride suppresses colorectal cancer cell survival and proliferation through ROS/GSK-3β/NF-κB signaling pathway. Oxid. Med. Cell Longev 2014, 241864–241864. 10.1155/2014/241864 25002914 PMC4070474

[B44] Li L. Peng X. Qin Y. Wang R. Tang J. Cui X. (2017a). Acceleration of bone regeneration by activating Wnt/β-catenin signalling pathway via lithium released from lithium chloride/calcium phosphate cement in osteoporosis. Sci. Rep. 7, 45204. 10.1038/srep45204 28338064 PMC5364554

[B45] Li L. Wang R. Li B. Liang W. Pan H. Cui X. (2017b). Lithium doped calcium phosphate cement maintains physical mechanical properties and promotes osteoblast proliferation and differentiation. J. BioMed. Mater. Res. B. Appl. Biomater. 105 (5), 944–952. 10.1002/jbm.b.33625 26856256

[B41] Li D. Xie X. Yang Z. Wang C. Wei Z. Kang P. (2018). Enhanced bone defect repairing effects in glucocorticoid-induced osteonecrosis of the femoral head using a porous nano-lithium-hydroxyapatite/gelatin microsphere/erythropoietin composite scaffold. Biomater. Sci. 6 (3), 519–537. 10.1039/C7BM00975E 29369309

[B46] Ling Z. Wu L. Shi G. Chen L. Dong Q. (2017). Increased Runx2 expression associated with enhanced Wnt signaling in PDLLA internal fixation for fracture treatment. Exp. Ther. Med. 13 (5), 2085–2093. 10.3892/etm.2017.4216 28565812 PMC5443172

[B48] Liu T. Zhang L. Joo D. Sun S.-C. (2017). NF-κB signaling in inflammation. Signal Transduct. Target Ther. 2, 17023. 10.1038/sigtrans.2017.23 29158945 PMC5661633

[B49] Liu W. Chen D. Jiang G. Li Q. Wang Q. Cheng M. (2018). A lithium-containing nanoporous coating on entangled titanium scaffold can enhance osseointegration through Wnt/beta-catenin pathway. Nanomedicine 14 (1), 153–164. 10.1016/j.nano.2017.09.006 28965979

[B47] Liu B. Wu Q. Zhang S. Del Rosario A. (2019). Lithium use and risk of fracture: a systematic review and meta-analysis of observational studies. Osteoporos Int. 30 (2), 257–266. 10.1007/s00198-018-4745-9 30374598

[B50] Loiselle A. E. Lloyd S. A. Paul E. M. Lewis G. S. Donahue H. J. (2013). Inhibition of GSK-3beta rescues the impairments in bone formation and mechanical properties associated with fracture healing in osteoblast selective connexin 43 deficient mice. PloS One 8 (11), e81399. 10.1371/journal.pone.0081399 24260576 PMC3832658

[B51] Luo J. Yang Z. Ma Y. Yue Z. Lin H. Qu G. (2016). LGR4 is a receptor for RANKL and negatively regulates osteoclast differentiation and bone resorption. Nat. Med. 22 (5), 539–546. 10.1038/nm.4076 27064449

[B52] Ma Y. Li Y. Hao J. Ma B. Di T. Dong H. (2019). Evaluation of the degradation, biocompatibility and osteogenesis behavior of lithium-doped calcium polyphosphate for bone tissue engineering. BioMed. Mater. Eng. 30 (1), 23–36. 10.3233/BME-181030 30530956

[B53] Mak T. W. Shek C. C. Chow C. C. Wing Y. K. Lee S. (1998). Effects of lithium therapy on bone mineral metabolism: a two-year prospective longitudinal study. J. Clin. Endocrinol. Metab. 83 (11), 3857–3859. 10.1210/jc.83.11.3857 9814458

[B54] Mason J. J. Williams B. O. (2010). SOST and DKK: Antagonists of LRP Family Signaling as Targets for Treating Bone Disease. J. Osteoporos 2010, 9. 10.4061/2010/460120 PMC2951123 20948575

[B55] McGonnell I. M. Grigoriadis A. E. Lam E. W. F. Price J. S. Sunters A. (2012). A specific role for phosphoinositide 3-kinase and AKT in osteoblasts? Front. Endocrinol. 3, 88–88. 10.3389/fendo.2012.00088 PMC3400941 22833734

[B56] Meehan A. D. Udumyan R. Kardell M. Landén M. Järhult J. Wallin G. (2018). Lithium-associated hypercalcemia: pathophysiology, prevalence, management. World J. Surg. 42 (2), 415–424. 10.1007/s00268-017-4328-5 29260296 PMC5762804

[B57] Moon J. B. Kim J. H. Kim K. Youn B. U. Ko A. Lee S. Y. (2012). Akt induces osteoclast differentiation through regulating the GSK3β/NFATc1 signaling cascade. J. Immunol. 188 (1), 163–169. 10.4049/jimmunol.1101254 22131333

[B58] Muneer A. (2017). Wnt and GSK3 Signaling Pathways in Bipolar Disorder: Clinical and Therapeutic Implications. Clin. Psychopharmacol. Neurosci. 15 (2), 100–114. 10.9758/cpn.2017.15.2.100 28449557 PMC5426498

[B59] Nair C. G. Menon R. Jacob P. Babu M. (2013). Lithium-induced parathyroid dysfunction: A new case. Indian J. Endocrinol. Metab. 17 (5), 930–932. 10.4103/2230-8210.117223 24083184 PMC3784886

[B60] Neelakandan R. S. Bhargava D. (2012). Transport distraction osteogenesis for maxillomandibular reconstruction: current concepts and applications. J. Maxillofac. Surg. 11 (3), 291–299. 10.1007/s12663-011-0329-3 PMC3428445 23997479

[B61] Nordenstrom J. Elvius M. Bagedahl-Strindlund M. Zhao B. Torring O. (1994). Biochemical hyperparathyroidism and bone mineral status in patients treated long-term with lithium. Metabolism 43 (12), 1563–1567. 10.1016/0026-0495(94)90017-5 7990712

[B62] Park J. H. Lee N. K. Lee S. Y. (2017). Current Understanding of RANK Signaling in Osteoclast Differentiation and Maturation. Mol. Cells 40 (10), 706–713. 10.14348/molcells.2017.0225 29047262 PMC5682248

[B63] Pepersack T. Corvilain J. Bergmann P. (1994). Effects of lithium on bone resorption in cultured foetal rat long-bones. Eur. J. Clin. Invest. 24 (6), 400–405. 10.1111/j.1365-2362.1994.tb02183.x 7957493

[B64] Pinna M. Manchia M. Puddu S. Minnai G. Tondo L. Salis P. (2017). Cutaneous adverse reaction during lithium treatment: a case report and updated systematic review with meta-analysis. Int. J. Bipolar Disord. 5 (1), 20–20. 10.1186/s40345-017-0091-7 28405955 PMC5495819

[B65] Plenge P. Rafaelsen O. J. (1982). Lithium effects on calcium, magnesium and phosphate in man: effects on balance, bone mineral content, faecal and urinary excretion. Acta Psychiatr. Scand. 66 (5), 361–373. 10.1111/j.1600-0447.1982.tb06718.x 6817593

[B66] Poels E. M. P. Bijma H. H. Galbally M. Bergink V. (2018). Lithium during pregnancy and after delivery: a review. Int. J. Bipolar Disord. 6 (1), 26–26. 10.1186/s40345-018-0135-7 30506447 PMC6274637

[B67] Rady A. Elsheshai A. Elkholy O. Abouelwafa H. Eltawil M. (2018). Long Term Use of Antipsychotics and Adverse Effects on Bone Density. 10.4172/Neuropsychiatry.1000491

[B68] Rybakowski J. K. (2018). Challenging the Negative Perception of Lithium and Optimizing Its Long-Term Administration. Front. Mol. Neurosci. 11, 349–349. 10.3389/fnmol.2018.00349 30333722 PMC6175994

[B69] Schrauzer G. N. (2002). Lithium: occurrence, dietary intakes, nutritional essentiality. J. Am. Coll. Nutr. 21 (1), 14–21. 10.1080/07315724.2002.10719188 11838882

[B70] Setiawati R. Rahardjo P. (2018). Bone Development and Growth *Osteogenesis and Bone Regeneration*: IntechOpen. 10.5772/intechopen.82452

[B71] Shi X. Wang J. Lei Y. Cong C. Tan D. Zhou X. (2019). Research progress on the PI3K/AKT signaling pathway in gynecological cancer (Review). Mol. Med. Rep. 19 (6), 4529–4535. 10.3892/mmr.2019.10121 30942405 PMC6522820

[B72] Soares-Silva M. Diniz F. F. Gomes G. N. Bahia D. (2016). The Mitogen-Activated Protein Kinase (MAPK) Pathway: Role in Immune Evasion by Trypanosomatids. Front. Microbiol. 7, 183–183. 10.3389/fmicb.2016.00183 26941717 PMC4764696

[B73] Stewart S. Bryant S. J. Ahn J. Hankenson K. D. (2015). “Chapter 24 - Bone Regeneration,” in Translational Regenerative Medicine. Eds. Atala A. Allickson J. G. (Boston: Academic Press), 313–333.

[B74] Su J. A. Cheng B. H. Huang Y. C. Lee C. P. Yang Y. H. Lu M. L. (2017). Bipolar disorder and the risk of fracture: A nationwide population-based cohort study. J. Affect. Disord. 218, 246–252. 10.1016/j.jad.2017.04.037 28477503

[B75] Tevlin R. McArdle A. Chan C. K. F. Pluvinage J. Walmsley G. G. Wearda T. (2014). Osteoclast derivation from mouse bone marrow. J. Vis. Exp. (93), e52056–e52056. 10.3791/52056 25407120 PMC4353410

[B76] Tondo L. Alda M. Bauer M. Bergink V. Grof P. Hajek T. (2019). Clinical use of lithium salts: guide for users and prescribers. Int. J. Bipolar Disord. 7 (1), 16–16. 10.1186/s40345-019-0151-2 31328245 PMC6643006

[B77] Vachhani K. Pagotto A. Wang Y. Whyne C. Nam D. (2018a). Design of experiments confirms optimization of lithium administration parameters for enhanced fracture healing. J. Biomech. 66, 153–158. 10.1016/j.jbiomech.2017.09.043 29162229

[B78] Vachhani K. Whyne C. Wang Y. Burns D. M. Nam D. (2018b). Low-dose lithium regimen enhances endochondral fracture healing in osteoporotic rodent bone. J. Orthop. Res. 36 (6), 1783–1789. 10.1002/jor.23799 29106746

[B79] Vestergaard P. Rejnmark L. Mosekilde L. (2005). Reduced relative risk of fractures among users of lithium. Calcif. Tissue Int. 77 (1), 1–8. 10.1007/s00223-004-0258-y 16007481

[B80] Walker R. J. Weggery S. Bedford J. J. McDonald F. J. Ellis G. Leader J. P. (2005). Lithium-induced reduction in urinary concentrating ability and urinary aquaporin 2 (AQP2) excretion in healthy volunteers. Kidney Int. 67 (1), 291–294. 10.1111/j.1523-1755.2005.00081.x 15610254

[B81] Wang H. Brown J. Martin M. (2011). Glycogen synthase kinase 3: a point of convergence for the host inflammatory response. Cytokine 53 (2), 130–140. 10.1016/j.cyto.2010.10.009 21095632 PMC3021641

[B82] Wang X. Zhu S. Jiang X. Li Y. Song D. Hu J. (2015). Systemic administration of lithium improves distracted bone regeneration in rats. Calcif. Tissue Int. 96 (6), 534–540. 10.1007/s00223-015-0004-7 25903228

[B83] Wei W. Zeve D. Suh J. M. Wang X. Du Y. Zerwekh J. E. (2011). Biphasic and dosage-dependent regulation of osteoclastogenesis by β-catenin. Mol. Cell Biol. 31 (23), 4706–4719. 10.1128/MCB.05980-11 21876000 PMC3232928

[B84] Wilting I. de Vries F. Thio B. M. Cooper C. Heerdink E. R. Leufkens H. G. (2007). Lithium use and the risk of fractures. Bone 40 (5), 1252–1258. 10.1016/j.bone.2006.12.055 17258948

[B85] Wong S. K. Mohamad N. V. Ibrahim N. Chin K. Y. Shuid A. N. Ima-Nirwana S. (2019). The Molecular Mechanism of Vitamin E as a Bone-Protecting Agent: A Review on Current Evidence. Int. J. Mol. Sci. 20 (6). 10.3390/ijms20061453 PMC6471965 30909398

[B86] Wu M. Chen G. Li Y.-P. (2016). TGF-β and BMP signaling in osteoblast, skeletal development, and bone formation, homeostasis and disease. Bone Res. 4, 16009–16009. 10.1038/boneres.2016.9 27563484 PMC4985055

[B88] Yang Y. Kim S. C. Yu T. Yi Y.-S. Rhee M. H. Sung G.-H. (2014). Functional roles of p38 mitogen-activated protein kinase in macrophage-mediated inflammatory responses. Mediators Inflammation 2014, 352371–352371. 10.1155/2014/352371 PMC3977509 24771982

[B87] Yang C. Wang W. Zhu K. Liu W. Luo Y. Yuan X. (2019). Lithium chloride with immunomodulatory function for regulating titanium nanoparticle-stimulated inflammatory response and accelerating osteogenesis through suppression of MAPK signaling pathway. Int. J. Nanomed. 14, 7475–7488. 10.2147/IJN.S210834 PMC6750619 31571859

[B89] Yavropoulou M. P. Yovos J. G. (2008). Osteoclastogenesis–current knowledge and future perspectives. J. Musculoskelet. Neuronal Interact. 8 (3), 204–216. 18799853

[B90] Zamani A. Omrani G. R. Nasab M. M. (2009). Lithium’s effect on bone mineral density. Bone 44 (2), 331–334. 10.1016/j.bone.2008.10.001 18992857

[B91] Zhai X. Wang S. Zhu M. He W. Pan Z. Su S. (2019). Antiviral Effect of Lithium Chloride and Diammonium Glycyrrhizinate on Porcine Deltacoronavirus *In Vitro* . Pathogens 8 (3), 144. 10.3390/pathogens8030144 PMC6789623 31505777

[B92] Zhang J. Cai L. Tang L. Zhang X. Yang L. Zheng K. (2018a). Highly dispersed lithium doped mesoporous silica nanospheres regulating adhesion, proliferation, morphology, ALP activity and osteogenesis related gene expressions of BMSCs. Colloids Surf. B. Biointerfaces 170, 563–571. 10.1016/j.colsurfb.2018.06.038 29975904

[B93] Zhang J. Cai L. Wang T. Tang S. Li Q. Tang T. (2018b). Lithium doped silica nanospheres/poly(dopamine) composite coating on polyetheretherketone to stimulate cell responses, improve bone formation and osseointegration. Nanomedicine 14 (3), 965–976. 10.1016/j.nano.2018.01.017 29408735

[B94] Zhou C. Fang L. Chen Y. Zhong J. Wang H. Xie P. (2018). Effect of selective serotonin reuptake inhibitors on bone mineral density: a systematic review and meta-analysis. Osteoporos. Int. : A J. established as result cooperation between Eur. Foundation Osteoporos. Natl. Osteoporos. Foundation U.S.A. 29 (6), 1243–1251. 10.1007/s00198-018-4413-0 29435621

[B95] Zomorodian E. Baghaban Eslaminejad M. (2012). Mesenchymal stem cells as a potent cell source for bone regeneration. Stem Cells Int. 2012, 980353–980353. 10.1155/2012/980353 22448175 PMC3289837

